# Proposal for a New Method for Evaluating Polymer-Modified Bitumen Fatigue and Self-Restoration Performances Considering the Whole Damage Characteristic Curve

**DOI:** 10.3390/polym16192782

**Published:** 2024-09-30

**Authors:** Songtao Lv, Dongdong Ge, Shihao Cao, Dingyuan Liu, Wenhui Zhang, Cheng-Hui Li, Milkos Borges Cabrera

**Affiliations:** 1 School of Traffic and Transportation Engineering, Changsha University of Science and Technology, Changsha 410114, China; lst@csust.edu.cn (S.L.); dge1@csust.edu.cn (D.G.); csh@stu.csust.edu.cn (S.C.); nf@stu.csust.edu.cn (D.L.); whzz@stu.csust.edu.cn (W.Z.); 2 State Key Laboratory of Coordination Chemistry, School of Chemistry and Chemical Engineering, Collaborative Innovation Center of Advanced Microstructures, Nanjing University, Nanjing 210093, China; chli@nju.edu.cn

**Keywords:** bitumen fatigue performance, failure definition, self-healing polymers, self-restoration performance, self-healing behavior

## Abstract

Fatigue performance and self-repairing activity of asphalt binders are two properties that highly influence the fatigue cracking response of asphalt pavement. There are still numerous gaps in knowledge to fill linked with these two characteristics. For instance, current parameters fail to accommodate these two bitumen phenomena fully. This study aims to propose a new procedure to address this issue utilizing the linear amplitude sweep (LAS) test, LAS with rest period (RP) (LASH) test, and simplified viscoelastic continuum damage (S-VECD) model. This research work used four different types of asphalt binders: neat asphalt (NA), self-healing thermoplastic polyurethane (STPU)-modified bitumen (STPB), self-healing poly (dimethyl siloxane) crosslinked with urea bond (IPA1w)-modified bitumen (IPAB), and styrene–butadiene–styrene (SBS)-modified bitumen (SBSB). Before the testing process, all the materials were subjected to short-term and long-term aging. The new procedure showed a superior capacity to analyze and accommodate all bitumen fatigue performances and self-repairing activities compared to the current method. Another finding proved that asphalt binders with a higher self-restoration behavior failed to show a better fatigue performance. Moreover, the higher fatigue performance increments produced by STPU and IPA1w in NA concerning the control bitumen were 123.7% and 143.7%, respectively. Those values were obtained with 1.0% STPU and 0.5% IPA1w in NA. A breakthrough finding demonstrated that asphalt binder fatigue response is augmented when the RP was applied at a higher damage intensity (*S*) value. STPB and IPAB reached their highest increments of fatigue response, containing 1.0% of STPU and 0.5% of IPA1w, respectively. Those augmentations were 207.54% and 232.64%, respectively.

- This is the only research project that utilizes a room-temperature self-healing polymer to modified bitumen.
- This is the only research project that proves the existence of a higher cumulative dissipated energy when applying the rest period at a higher damage level.
- This is the only research project that proves the room-temperature self-healing polymer can improve the fatigue performance of asphalt binder.
- Comment that the error bar is smaller in %ξ than in %R.

## 1. Introduction

The fatigue cracking phenomenon is a typical damage found in asphalt pavement, and it represents one of the main reasons for the distress and failures of this type of road structure element [1,2,3]. The load from vehicle traffic and climate change are the factors with the strongest influences on the occurrence of fatigue cracking, which proliferates in asphalt mixtures throughout the bituminous material phase [4]. Therefore, the fatigue cracking responses of any asphalt mixture and pavement notably depend on the fatigue cracking behavior of bituminous materials [5,6,7].

The self-healing characteristics of asphalt binders can help reduce fatigue cracking in asphalt mixtures and pavement. However, there are numerous gaps in knowledge to understand and promote this property fully. As a result, countless research works have studied this phenomenon [8]. Currently, two effective tools exist to appraise the fatigue cracking response and self-healing activity of asphalt binder. These tools are the Linear amplitude sweep (LAS) test (AASHTO TP 101) and the simplified viscoelastic continuum damage (S-VECD) method [9]. Complex shear modulus, shear stress, shear strain, and phase angle are four of the most critical parameters obtained from the LAS test. Then, those parameters are processed using the S-VECD theory, to determine the material integrity (C) and *S* values. Subsequently, the fatigue performance and self-healing activity can be evaluated and predicted [10,11,12]. The *S* parameter represents the internal state condition of the bitumen and can be determined by applying the damage evolution of Schapery’s work potential theory (see Equation (1)). Afterward, the corresponding *C* and *S* values can be plotted in a *C* vs. *S* graph for building the damage characteristic curve (DCC) [13,14]:

(1) $$\frac{dS}{d\vartheta }={\left(\frac{{\partial W}^{R}}{\partial S}\right)}^{\alpha },$$

where α, ϑ, and $W^{R}$ are damage evolution rate, reduced time, and pseudo-strain energy, respectively.

Self-healing in asphalt binders (including asphalt mixtures and asphalt pavements) is mainly influenced by molecular interdiffusion and capillary flow [15]. The former parameter has a decisive role in the self-healing process of asphalt. The existence of high temperature promotes molecular interdiffusion because it increases the kinetic energy and bitumen molecular movement. Moreover, the density of the asphalt binder and its glass transition temperature are critical factors of molecular interdiffusion [15,16]. The occurrence of fatigue cracking happens at the final stage of pavement service life; this is the period when the asphalt binders are stiff and rigid. The asphalt binder at the end of the pavement service life has been subjected to a long-term aging process, mainly by receiving the cyclic load of traffic, as mentioned before. At this stage, the horizontal tensile strains at the pavement layer bottom reach a greater value than pavement tensile strength characteristics. This phenomenon causes microcracks [17]. Then, these cracks spread and increase their size (length and width), creating serious damage to pavement structure [18].

Various research works have evaluated the fatigue performance of asphalt mixtures and types of bitumen using the previous framework and others. Jiao et al. [19] appraised the fatigue behavior of asphalt mixture and fine aggregate matrix mixes containing reclaimed asphalt pavement. This research team set a correlation between fatigue life ($N_{f}$ = number of loading cycles to reach bitumen failure point) and strain level linked with both mentioned materials. The obtained LAS experimental data in this study, linked with the fine aggregate matrix mixes, exhibited an acceptable agreement with the four-point beam fatigue testing outcomes. Muhammad et al. [20] tried to link the LAS test results of asphalt binders with the indirect tensile test, semi-circular bending test, and four-point bending beam test results using statistical tools. This study found that the secant modulus associated with the semi-circular bending test is the parameter that best correlates with bitumen properties and asphalt mixture properties. The LAS test results illustrated a superior capacity to model the four-point bending beam test results.

Sabouri et al. [21] proved the existence of a high relationship between LAS test results linked with asphalt binder and the fatigue data of the four-point bending beam test associated with asphalt mixture at different strain levels. Moreover, this research work demonstrated that the bitumen fatigue index (G*sinδ) correlated poorly with the LAS and four-point bending beam fatigue tests. Ilyin and Yadykova [22] utilized the Glover–Rowe parameter to evaluate the performance of various polymer-modified bitumen. This parameter, in combination with the time–temperature superposition principle, allowed these researchers to quickly estimate the fatigue cracking and rutting resistances of the polymer-modified bitumen at low and high temperatures.

Numerous studies have assessed the self-healing capacities of neat asphalt binders (NA) and polymer-modified bitumen. Xie et al. [23] evaluated the self-healing capabilities of NA and SBSB by utilizing LAS, LASH, and S-VECD methods. This research team introduced the percent healing (%Hs) parameter and the rest-damage superposition principle to build the %Hs mastercurve. This study found a superior %Hs in NA than in SBSB. Wang et al. [24] appraised the NA and SBSB self-healing performances after short- and long-term aging processes by using LASH, VECD, and %Hs mastercurve. In addition, this research work correlated the bitumen composition with self-healing behavior. This study identified two factors that diminished bitumen self-healing activity: the aging process and SBS. However, light fractions, small molecules, and longer molecules augmented the bitumen’s self-healing performance.

Wang et al. [9] assessed the self-healing activity of NA and SBSB by conducting LASH, VECD, and microstructure tests. Researchers found that a superior number of saturates/aromatic fractions and small molecules promoted the bitumen self-healing behavior. SBSB and NA exhibited similar self-healing activity, which is not in line with the findings of Xie et al. [23] and Wang et al. [24]. Aurilio [25] and Aurilio and Baaj [26] analyzed the performances of self-healing polymer-modified bitumen (SPB) and SBSB by utilizing LASH, a simplified LASH, and S-VECD methods. Researchers found that the elastomeric properties of bitumen improved by adding the self-healing polymer, but this material could not promote the self-healing capability in the asphalt binder. In addition, SBS stimulated the crack healing activity in the bitumen, which is in the opposite direction of the findings in Xie et al. [23] and Wang et al. [24].

Aurilio et al. [27] assessed the performances of chemical warm mix additive-modified bitumen (WMAB) and NA by utilizing simplified LASH and S-VECD methods. This study found that aged NA exhibited superior self-healing behavior than aged WMAB. Almutairi and Baaj [28] appraised the behavior of NA, SBSB, glass powder-modified bitumen (GPB), glass powder and phase-change material(GPCM)-modified bitumen (GPCMB) by conducting LASH and S-VECD methods. This study found that GPCM maintained a similar and improved self-healing capacity of base asphalt when 5 and 30 min of RP were applied, respectively. In addition, glass powder increased and reduced the self-healing performance of NA and SBSB, respectively.

Lv et al. [29] analyzed the self-healing performances of NA and STPB (which utilized STPU: a room-temperature [25 °C] self-reinforcing, self-healing polymer). This study utilized LASH and S-VECD in the testing process. Researchers found that STPU promoted the self-healing performance of base asphalt. Furthermore, higher $N_{f}$ and %Hs could not ensure higher fatigue performance; hence, the current framework could not fully accommodate the self-healing behavior of asphalt binders. As a result, a new procedure was proposed considering the area below the DCC.

All previous research works on bitumen self-healing analysis and fatigue assessment utilized the peak of stored pseudo-strain energy (PSE) to define $N_{f}$. As mentioned before, this concept identifies the needed number of loading cycles (N) to reach the bitumen failure point. However, Lv et al. [29] and Wang et al. [10] proved the existence of ranking inconsistency between $N_{f}$ and a DCC analysis in terms of asphalt binder fatigue performance. Hence, all previous studies reached conclusions based on a concept with inadequacy issues. As a result, Lv et al. [30] introduced a new framework based on S instead of N to overcome the abovementioned inconsistency. The new framework used the stored potential cohesion (SPC) peak (see definition in Section 2.10) as the failure definition. This research work tested NA, STPB, SBSB, and IPAB (which utilized IPA1w: a room-temperature [25 °C] self-healing polymer). Table 1 summarizes the main studies on bitumen self-healing and fatigue performances.

As can be seen in Table 1, currently, there is no framework to evaluate bitumen fatigue performances with the following capacities simultaneously:

1. A framework to ensure higher fatigue performance in terms of a DCC analysis for bitumen with higher %Hs when assessing the fatigue behavior of a group of asphalt binders.
2. A framework to ensure higher fatigue performance in terms of a DCC analysis for bitumen with a higher failure definition when analyzing the fatigue response of various bituminous materials.
3. A framework to ensure ranking consistency between the bitumen failure definition and its fatigue performance in terms of a DCC analysis when appraising the fatigue behavior of numerous asphalt binders.

Engineers play a crucial role in selecting the most convenient asphalt binder for a specific road construction project. A framework that simultaneously exhibits the three mentioned capacities can empower them to make the best choice. However, the current scenario confirms that, when using the available frameworks to evaluate bitumen fatigue performance (including self-restoration activity), there is a low likelihood of selecting the most proper bituminous material for a specific road project. This phenomenon is because the available frameworks fail to ensure the three abovementioned capacities to assess the fatigue response of bituminous materials. As a result, a framework that simultaneously exhibits the three mentioned capacities can empower engineers to select the most convenient asphalt binder for any road project and ensure the successful service life of the asphalt pavement [12].

Hence, this study will introduce a composite procedure with the three abovementioned capacities simultaneously to ensure the successful selection of the most convenient asphalt binder among a group of bituminous materials for any road construction project. The newly proposed composite framework will be based on S instead of N because Lv et al. [29] and Lv et al. [30] demonstrated that S is more capable of modeling the fatigue behavior of asphalt binders than N. As a result, this study represents a continuation of these two published manuscripts.

The efficiency of the composite procedure will be thoroughly verified by testing eight different types of bitumen. This study, however, takes a unique approach by testing the capacity of two innovative room-temperature (25 °C) self-healing polymers (STPU and IPA1w) to promote the self-restoration activity of NA. These polymers, unlike others, do not need extra stimuli (microwave and induction heating) to incentivize their self-healing behavior. This unique property could be a game-changer for asphalt pavement surface and layers because this special characteristic promotes self-restoration activity in an asphalt pavement structure. Hence, this study may define a new generation of asphalt binder modifiers, a concept that has not been explored before. The only previous experience with this specific type of polymer can be found in the following research works: Lv et al. [29] and Lv et al. [30]. These published manuscripts belong to the same big project of this study that this research team is carrying out.

## 2. Materials and Methods

### 2.1. Neat Asphalt

This study purchased and utilized 70# NA from China Petroleum & Chemical Corporation, Jinling Branch in Nanjing City (Jiangsu Province, China). This type of NA is popular in China and it has prominently exhibited an excellent capacity for withstanding the traffic loading cycle [31]. NA’s physical properties (see Table 2) observe the standard required values in Chinese specifications. These documents are based on AASHTO and American Society for Testing and Materials standard specifications.

### 2.2. Styrene–Butadiene–Styrene-Modified Bitumen

SBS is an asphalt binder modifier that increases NA’s rutting resistance, cohesion, adhesion, and elasticity properties [32]. Accordingly, SBS has become a popular bitumen modifier to produce SBSB, which is globally used for road construction [33]. Hence, this study included SBSB as bitumen to test; Table 3 illustrates its physical properties.

Gel permeation chromatography was used to determine the molecular weight of the SBSB, and the type of molecular weight was the average molecular weight.

### 2.3. Self-Healing Thermoplastic Polyurethane

This research work continues two studies mentioned above: Lv et al. [29] and Lv et al. [30]. Both studies utilized STPU. Hence, this research team decided to include STPU in this new round of experiments, which Nanjing University elaborated.

The material components utilized in the synthesis of the STPU are as follows: polytetramethylene ether glycol (Mn = 1000 g/mol, f = 2), the catalyst dibutyltin dilaurate (DBTDL), and chain extender 3-Dimethylaminopropylamine, which were purchased from Aladdin. Adams Enterprise sent us the isophorone di-isocyanate. All these substances were used without a further purification process. Tetrahydrofuran (Sigma-Aldrich, St. Louis, MO, USA) and chloroform (CHCl_3_, Sigma-Aldrich) were used after CaH_2_ redistillation.

A crystallizable soft segment (polytetramethylene ether glycol) is one of the main components of STPU, and its meticulously selected length ensures a lower crystallization energy threshold when subjecting the STPU to the stretching process in the elongation test. Stratified H-bonding interactions are named bonds with sacrificial and active properties, guaranteeing hard domains with as low as possible binding energy characteristics. This fact raises the probability that hard domain segments connect with small-sized hard domains via H-bonding. As a result, the self-healing activity is promoted without extra stimuli (microwave and heat). This phenomenon is appropriate for mitigating crack occurrence on road surfaces. Furthermore, a strain-induced crystallization property of STPU ensures a retarded but reversible self-reinforcing effect [34]

Table 4 illustrates the physical properties of STPU. For more details on this novel polymer, readers can see Li et al. [34], Lv et al. [29], and Lv et al. [30].

Gel permeation chromatography was used to determine the molecular weight of the STPU, and the type of molecular weight was the average molecular weight.

### 2.4. Self-Healing Poly(dimethyl siloxane) Crosslinked with Urea Bond

As mentioned before, this research work is a continuation of previous studies by Lv et al. [29] and Lv et al. [30]. The latter study introduced IPA1w because it exhibited suitable properties for road construction. As a result, this research team included the IPA1w in this new group of tests, and Nanjing University produced it. In addition, IPA1w is a room-temperature (25 °C) self-healing polymer that promotes self-healing behavior without extra stimuli (microwave and heat). This property could be proper for road surfaces.

The substance components utilized in synthesizing IPA1w are Bis(3-aminopropyl)-terminated PDMS (Mn = 10,000 g mol^−1^, noted as A1w) received from Gelest. This study purchased isophorone di-isocyanate from Sigma-Aldrich and further distilled tetrahydrofuran for use later.

The synthesis of IPA1w occurred as follows: The redistilled tetrahydrofuran (100 mL) and the A1w (4.00 g, 0.4 mmol) were mixed and continuously stirred in an ice bath for 30 min. Then, 30 mL of tetrahydrofuran was slowly mixed with the solution of isophorone di-isocyanate (91.13 mg, 0.41 mmol) using a constant pressure funnel. These substances reacted, and the mixture was stirred for 24 h under an N_2_ atmosphere at room temperature until a concentrated sticky mucus was obtained. The resultant product was purified by utilizing cycles of the dissolution-precipitation-decantation process. The collected solution was then decanted into customized polytetrafluoroethylene molds and subjected to a drying process at 85 °C for 24 h. The obtained IPA1w polymer film was then peeled off for further testing.

The self-healing activity in IPA1w can be stimulated by breaking and relinking the hydrogen bonds and taking down and rebuilding the polymer chains at room temperature. The polymer units containing hydrogen bonds are more likely to join their chains and ensure entanglements [35]. Table 5 displays the physical properties of IPA1w. For more details associated with IPA1w, see Wang et al. [35] and Lv et al. [30].

Gel permeation chromatography was used to determine the molecular weight of the IPA1w, and the type of molecular weight was the average molecular weight.

### 2.5. Preparation Method of Self-Healing Thermoplastic Polyurethane-Modified Bitumen

The STPU and NA were mixed at 3500 revolutions per minute (shear speed), for 1 h (time), and at 170 °C (temperature) to elaborate the SPTB at laboratory levels. In addition, the 0.5, 1.0, and 1.5 wt% of STPU were the amounts of polymer mixed with NA to produce STPB0.5, STPB1.0, and STPB1.5, respectively. These mixing conditions and percentages of polymer were decided according to previous experience from Lv et al. [29] and Lv et al. [30], because these STPB showed superior fatigue performances in terms of a DCC assessment, self-healing capacities, and fatigue failure points regardless of the test conditions.

### 2.6. Preparation Method of Self-Healing Poly(dimethyl siloxane) Crosslinked with Urea Bond-Modified Bitumen

This study conducted the mixing process of IPA1w and NA setting the shear speed, time, and temperature at 3500 revolution per minute, 1 h, and 170 °C, respectively, to obtain IPAB. Furthermore, the 0.5, 1.0, and 1.5 wt% of IPA1w were the percentages of polymer mixed with NA to elaborate IPAB0.5, IPAB1.0, and IPAB1.5, respectively. These mixing conditions and amounts of polymers were decided considering the previous experience from Lv et al. [30] and Yang et al. [36], because these IPAB exhibited a higher self-restoration capacity, rutting resistance, and viscoelasticity.

### 2.7. Aging Procedure

This study used short-term and long-term aging procedures to cause the aging effect on NA, SBSB, STPB0.5, STPB1.0, STPB1.5, IPAB0.5, IPAB1.0, and IPAB1.5. All types of bitumen were subjected to both tests. The former process is described in the AASHTO T240 [37], and it is known as the rolling thin film oven (RTFO) test. The latter test is explained in the AASHTO R28-12 [38], and is known as the pressurized aging vessel (PAV) test.

### 2.8. Performance Grade (PG) Characterization Method

This research work conducted a group of tests to determine the PG of each type of bitumen. These unaged bitumen were subjected to a flash point temperature test (AASHTO T48-06) [39] and a rotational viscosity test (AASHTO T316) [40]. The RTFO-aged and unaged asphalt binders were tested to calculate the rutting index (AASHTO T315-20) [41]. RTFO+PAV-aged bitumen were subjected to fatigue cracking index (AASHTO T315-20) [41] and bending beam rheometer test (AASHTO T313-12) [42]. The PG for each bitumen was identified by utilizing AASHTO M320-10 [43]. The determined PGs are as follows: NA (PG 64-16), SBSB (PG 76-22), STPB0.5 (PG 64-22), STPB1.0 (PG 64-22), STPB1.5 (PG 64-16), IPAB0.5 (PG 64-22), IPAB1.0 (PG 64-16), and IPAB1.5 (PG 64-10).

### 2.9. Linear Amplitude Sweep (LAS) Test

In this study, the mastercurve and damage evolution rate “α” were determined by conducting the frequency sweep tests from 0.1 rad/s to 100 rad/s at different temperatures (20 °C, 25 °C, 30 °C, 35 °C, and 40 °C) with a strain level equal to 0.1%. The α value was determined as follows: α = 1/m + 1, where “m” is the mastercurve higher slope (absolute value) in a log–log graph [44]. Afterward, the effective procedure LAS test (AASHTO TP101) was carried out to analyze the intermedia temperature fatigue performance of RTFO + PAV aged bitumen. This test was carried out at 10 Hz, with the strain amplitude linearly increasing from 0.1% to 30% for 3100 cycles (standard value); this is called the continuous LAS test (cLAS) [23]. The parallel plate geometry and its gap were set in the dynamic shear rheometer at 8 mm and 2 mm, respectively, to appraise the asphalt binder performance of RTFO + PAV-aged bitumen. The temperature was set at 28 °C because, in a previous study (Lv et al. [30]), most of the time, all asphalt binders exhibited superior fatigue performances at this temperature.

### 2.10. Simplified Viscoelastic Continuum Damage (S-VECD) Model

The S-VECD model was utilized to analyze and process the LAS experimental data. This procedure determines *C* and *S* values, and its correlation is independent of loading history, regardless of the type of bitumen. As a result, different asphalt binder fatigue responses under any selected conditions with few experimental data can be determined [23,45,46]. After obtaining the *C*–*S* correlation (see Equation (2)), the DCC can be plotted in a *C* vs. *S* graph [29]. The *C* and Δ*S* (damage increment) values were calculated by utilizing Equations (3) and (4), respectively [44].

(2) $$C=1-C_{1}\cdot \left(S^{C_{2}}+C_{3}\right)\;with\;S=\sum_{i=1}^{S_{f}}{∆S}_{i},$$

(3) $$C=\frac{\left|G*\right|}{{\left|G*\right|}_{LVE}\;\cdot DMR}\;with\;DMR=\frac{{\left|G*\right|}_{fingerprint}}{{\left|G*\right|}_{LVE}},$$

(4) $$∆S_{i}={\left(\frac{1}{2}DMR\cdot {\left(\gamma_{i}^{R}\right)}^{2}\cdot \left(C_{i-1}-C_{i}\right)\right)}^{\frac{\alpha }{1+\alpha }}\cdot Q\;with\;Q\equiv {\left[\int {\left(sin\left(w_{r}\vartheta \right)\right)}^{2\alpha }d\vartheta \right]}^{\frac{1}{1+\alpha }},$$

In Equation (2), $C_{1}$, $C_{2}$, and $C_{3}$ are regression constants, and $S_{f}$ represents the *S* value at the failure point. For Equation (3), the parameters G*, ${\left|G*\right|}_{LVE}$, DMR, and ${\left|G*\right|}_{fingerprint}$ signify dynamic shear modulus (damaged), undamaged dynamic shear modulus (linear viscoelastic range), dynamic modulus ratio, and the initial dynamic shear modulus when conducting cLAS, respectively. In addition, the parameters $\gamma_{i}^{R}$, $w_{r}$, ϑ, and *i*-th in Equation (4) denote pseudo-strain amplitude, reduced angular frequency, reduced time, and the cycle of interest, respectively. The terms $W^{R}$ (stored PSE) and $\gamma_{i}^{R}$ are calculated by using Equations (5) and (6), respectively [44].

(5) $$W^{R}=\frac{1}{2}DMR\cdot C\left(S\right)\cdot {\left(\gamma^{R}\right)}^{2},$$

(6) $$\gamma_{i}^{R}\left(\vartheta \right)=\gamma_{i}\cdot {\left|G*\right|}_{LVE}\cdot sin\left(\omega_{r}\vartheta \right),$$

where $\gamma_{i}$ illustrates the shear strain amplitude in Equation (6). Lv et al. [30] introduced a new framework with a new concept of failure definition (which is the parameter that defines the fatigue life) that solved the ranking inconsistency issue between the traditional failure definition (stored PSE peak) and the DCC analysis in terms of bitumen fatigue performance. Accordingly, this research team utilized that new framework and new failure definition in this study.

**Figure 1:**
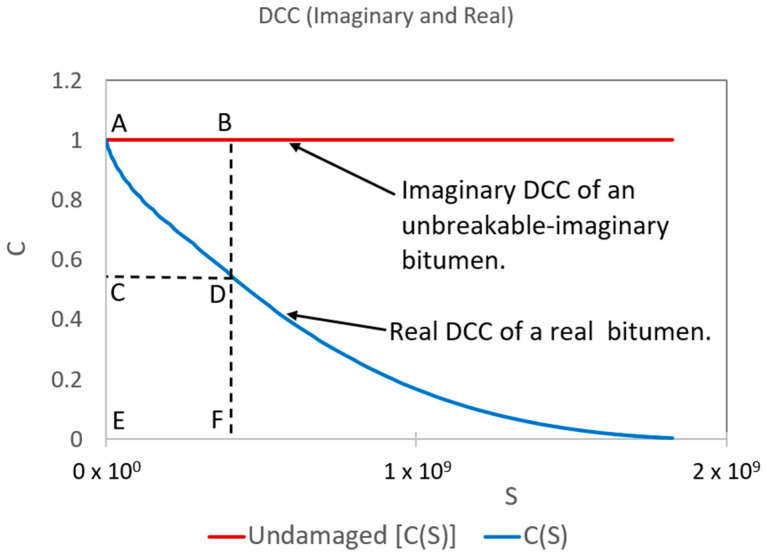
Representation of imaginary and real DCC to define TPC, SPC, and RPC.

The new framework mentioned above defined numerous new parameters, but there are three essential definitions: total potential cohesion (TPC), stored potential cohesion (SPC), and released potential cohesion (RPC). The TPC determines the imaginary bitumen strength capacity at each loading cycle to keep its *C* values equal to 1 while carrying out the cLAS procedure, even if damage has occurred. The TPC is represented by the imaginary rectangular area (A, B, F, and E) in Figure 1, and its formula is Equation (7). The SPC identifies the bitumen strength capacity at each loading cycle to maintain the *C* values as high as possible when conducting the cLAS test, even if damage has occurred. The rectangular area (C, D, F, and E) in Figure 1 depicts the SPC; its formulation is Equation (8). The RPC reveals the dissipated bitumen strength capacity at each loading cycle to uphold *C* values as high as possible while undertaking the cLAS test. The RPC is defined by the rectangular area (A, B, D, and C) in Figure 1, and its expression is Equation (9) [30].

(7) $${TPC}_{i}=S_{i}\cdot C_{0}\;\left(where\;C_{0}=1\right),$$

(8) $${SPC}_{i}=S_{i}\cdot C_{i},$$

(9) $${RPC}_{i}={TPC}_{i}-{SPC}_{i},$$

In Equation (7), ${TPC}_{i}$, $S_{i}$, and $C_{0}$ represent the total potential cohesion at the *i*-th cycle, the *S* value at the *i*-th cycle, and the constant material integrity equal to 1, respectively. In Equation (8), ${SPC}_{i}$ and $C_{i}$ are the stored potential cohesion and the *C* value at the i-th cycle, respectively. In the case of Equation (9), ${RPC}_{i}$ is the released potential cohesion at the *i*-th cycle [30].

**Figure 2:**
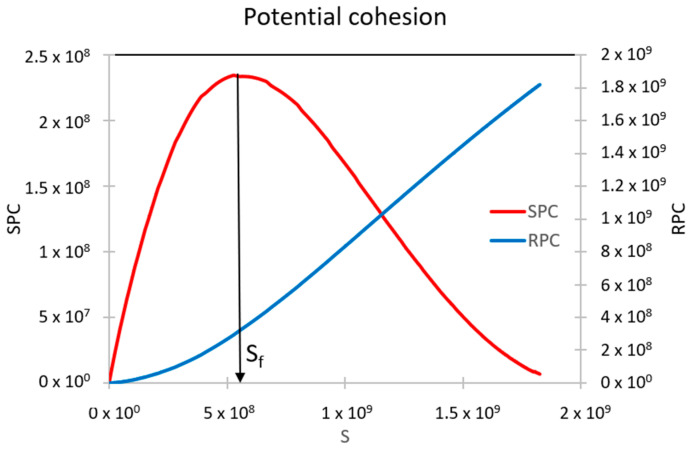
SPC and RPC curves.

Figure 2 illustrates the SPC and RPC curves. While the SPC curve increases, the asphalt binder maintains the strength capacity to store additional damage intensity when conducting the cLAS procedure. Nevertheless, when the SPC curve decreases, the bitumen fails to uphold the strength capacity to store additional damage intensity and, as a result, asphalt binder failure occurs. Hence, the peak of the SPC curve is considered the failure definition, and this new concept has the novelty that it is mainly based on S instead of N. Moreover, a higher SPC value means a superior fatigue response at the chosen loading cycle. The RPC curve grows from the starting point of the test (i.e., the asphalt binder loses strength capacity from the beginning) [30]. For more details about the new failure definition concept, see Lv et al. [30].

### 2.11. Linear Amplitude Sweep Test with Rest Period (LASH) (Traditional)

In this research work, the traditional LASH test considered the failure definition to be the peak of the SPC, instead of the peak of the stored PSE (the previous traditional failure definition). Lv et al. [30] proved that the locations of the SPC and stored PSE peaks on the stored PSE curve were similar, regardless of the asphalt binder, temperature, and aging conditions. This fact demonstrated that both definitions were compatible in identifying the failure point, even though their basements differed. This research team utilized the SPC peak as the failure definition because it eliminated the ranking inconsistency between the fatigue life (the traditional failure definition) and the DCC analysis regarding bitumen fatigue performance.

The LASH procedure was conducted with different RPs and damage levels (DLs). The former parameter was set at 1, 5, 15, and 30 min. The latter was set to be 25%, 50%, 75%, and 125% of $S_{f}$ (to test the bitumen before and after the failure point). These testing conditions were selected according to previous experience [23,27,29]. The conditions for the dynamic shear rheometer to carry out the LASH were the same as those for the LAS test, and the temperature was set at 28 °C.

**Figure 3:**
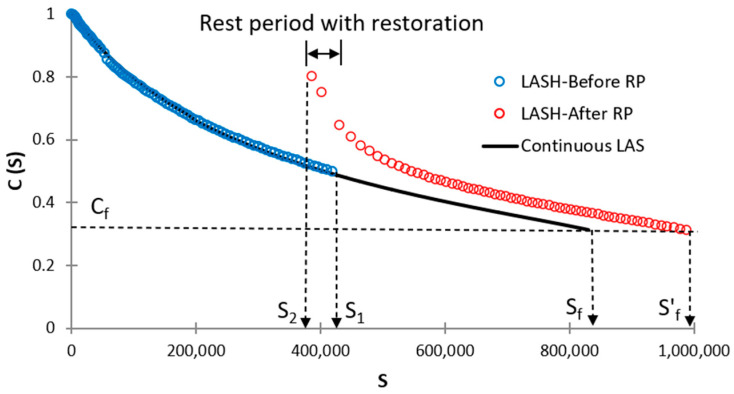
Graph to identify $S_{f}$ and $S_{f}^{\prime }$ when conducting LASH (RP before $S_{f}$).

Thixotropy is an important bitumen property for assessing its self-healing performance [47]. Currently, the LASH procedure determines restoration, as this concept was defined by Leegwater et al. [48]. The restoration includes “real” self-healing activity as a reversible phenomenon and thixotropy. As a result, this study utilized the term “percent restoration” (%R_s_) instead of percent healing, as introduced by Aurilio et al. [27]. The %R_s_ was determined as follows:

(10) $${\%R}_{s}=\frac{\left(S_{1}-S_{2}\right)}{S_{1}}\cdot 100,$$

where $S_{1}$ and $S_{2}$ are *S* values at the end of the first loading phase and at the starting point of the second loading phase, respectively (see Figure 3).

### 2.12. Linear Amplitude Sweep Test with Rest Period (LASH) (Updated)

The updated LASH test in this research work also considered the peak of the SPC curve, instead of the peak of the stored PSE curve (the previous traditional failure definition), as the failure definition.

The updated LASH procedure was conducted using the same RPs, DLs, and temperature utilized in the Section 2.11. This research work utilized the “percent restoration efficiency” (%ξ) concept, introduced by Lv et al. [29], to evaluate the self-restoration capacity and fatigue performance of bitumen at the same time. This concept was proposed by Lv et al. [29] because the research team identified that higher self-restoration capacity did not always ensure superior fatigue performance for asphalt binders in terms of a DCC analysis.

**Figure 4:**
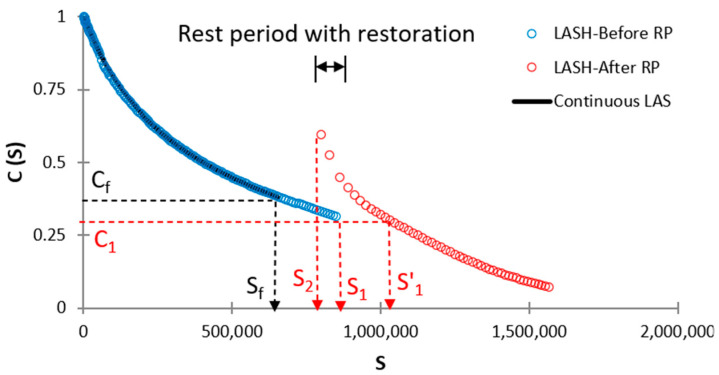
Graph to identify $S_{1}$ and $S_{1}^{\prime }$ when conducting LASH (RP after $S_{f}$).

The $\varphi^{p}$, $\varphi^{ph}$, $\varphi^{p\prime }$, and $\varphi^{ph\prime }$ parameters are named bitumen fatigue-potential performances up to $S_{f}$ (see Figure 3), $S_{f}^{\prime }$ (see Figure 3), $S_{1}$ (see Figure 4), and $S_{1}^{\prime }$ (see Figure 4), respectively. These parameters can be determined by obtaining the area under the DCC in each specific case of Figure 3 or 4. Equations (11)–(15) show how to calculate $\varphi^{p}$, $\varphi^{ph}$, $\varphi^{p\prime }$, $\varphi^{ph\prime }$, and %ξ, respectively.

(11) $$\varphi^{p}=\int_{0}^{S_{f}}1-C_{1}\cdot \left(S^{C_{2}}+C_{3}\right),$$

(12) $$\varphi^{ph}=\int_{0}^{S_{1}}1-C_{4}\cdot \left(S^{C_{5}}+C_{6}\right)+\int_{S_{2}}^{S_{f}^{\prime }}{-log}_{C_{7}}\left(S+C_{8}\right)+C_{9},$$

(13) $$\varphi^{p\prime }=\int_{0}^{S_{1}}1-C_{10}\cdot \left(S^{C_{11}}+C_{12}\right),$$

(14) $$\varphi^{ph\prime }=\int_{0}^{S_{1}}1-C_{13}\cdot \left(S^{C_{14}}+C_{15}\right)+\int_{S_{2}}^{S_{1}^{\prime }}{-log}_{C_{16}}\left(S+C_{17}\right)+C_{18},$$

(15) $$\%\xi =\left(\left(\varphi^{ph}-\varphi^{p}\right)/\varphi^{p}\right)\cdot 100\;or\;\%\xi =\left(\left(\varphi^{ph\prime }-\varphi^{p\prime }\right)/\varphi^{p\prime }\right)\cdot 100,$$

where $C_{4}$, $C_{5}$, $C_{6}$, $C_{7}$, $C_{8}$, $C_{9}$, $C_{10}$, $C_{11}$, $C_{12}$, $C_{13}$, $C_{14}$, $C_{15}$, $C_{16}$, $C_{17}$, and $C_{18}$ are regression constants; and $S_{2}$ represents the *S* value after the RP in both Figure 3 and Figure 4. A higher %ξ value means that the testing asphalt binder has superior fatigue performance concerning the fatigue-potential performance of the selected control bitumen. Accordingly, researchers can evaluate the asphalt binder fatigue performance by referencing any decided bitumen. The %ξ evaluates the bitumen fatigue behavior considering the effect of adding a polymer into NA, RP, and DL simultaneously. Readers can see Lv et al. [29] for more details about this procedure.

**Figure 5:**
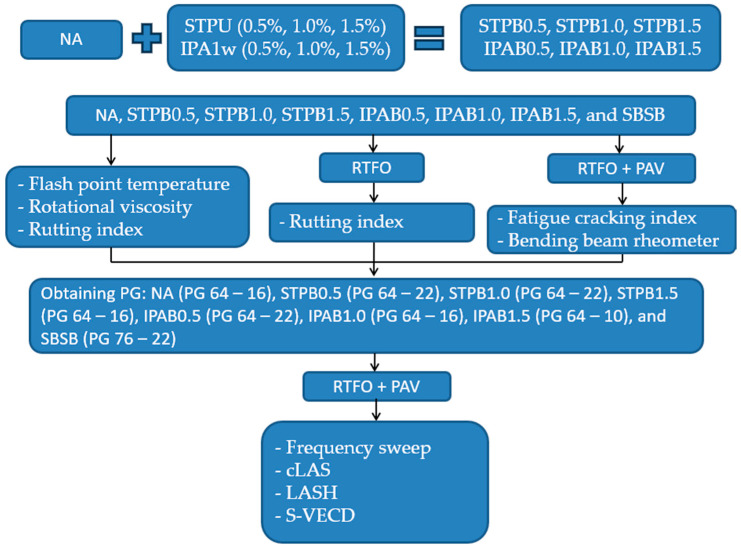
General flowchart of all procedures conducted in this research work.

Moreover, this study introduces a new concept to compare the cLAS test results of two types of bitumen of interest. The new parameter is named the “fatigue-potential performance increment (%β)” (see Equation (16)), and it is defined as the percent increment of $\varphi^{p}$ or $\varphi^{p\prime }$ related to one asphalt binder of interest concerning $\varphi^{p}$ or $\varphi^{p\prime }$ linked with a defined control bitumen. This parameter assesses the fatigue performance of a specific bitumen of interest concerning a decided control asphalt binder when subjected to the cLAS test. The %β parameter appraises the fatigue behavior of the bituminous materials only considering the effect of adding a polymer into NA (without considering the RP and DL).

(16) $$\%\beta =\left(\left(\varphi^{p_{1}}-\varphi^{p}\right)/\varphi^{p}\right)\cdot 100\;or\;\%\beta =\left(\left(\varphi^{p_{1}^{\prime }}-\varphi^{p^{\prime }}\right)/\varphi^{p^{\prime }}\right)\cdot 100,$$

where $\varphi^{p_{1}}$ and $\varphi^{p_{1}^{\prime }}$ are $\varphi^{p}$ values of the asphalt binders of interest when assessing the fatigue performance up to $S_{f}$ and after $S_{f}$, respectively. Hence, obtaining the difference between the corresponding %ξ and %β makes it possible to determine the fatigue performance of the bitumen of interest concerning a decided control asphalt binder only considering the effect of the RP and DL. This parameter is defined as the “fatigue-potential performance difference” (%δ) (see Equation (17). Figure 5 illustrates the general flowchart of all procedures carried out in this study.

(17) $$\%\delta =\left[\left(\frac{\varphi^{ph}-\varphi^{p}}{\varphi^{p}}\right)\cdot 100\right]-\left[\left(\frac{\varphi^{p_{1}}-\varphi^{p}}{\varphi^{p}}\right)\cdot 100\right]\;or\;\%\delta =\left[\left(\frac{\varphi^{ph\prime }-\varphi^{p\prime }}{\varphi^{p\prime }}\right)\cdot 100\right]-\left[\left(\frac{\varphi^{p_{1}^{\prime }}-\varphi^{p^{\prime }}}{\varphi^{p^{\prime }}}\right)\cdot 100\right]$$

## 3. Results and Discussion

### 3.1. Analysis of %R_s_ and %ξ (Respect to cLAS of PAV.SBSB) Values

**Figure 6:**
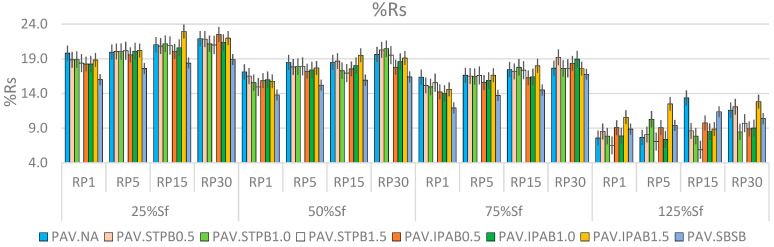
%R_s_ values linked with each bitumen at different DLs and RPs.

**Figure 7:**
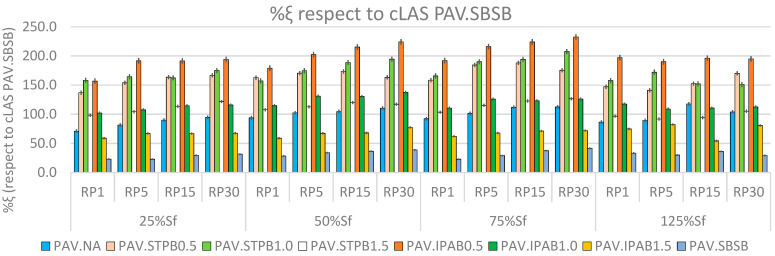
%ξ values related to each asphalt binder at different DLs and RPs.

Figure 6 and Figure 7 exhibit the %R_s_ and %ξ values associated with each asphalt binder in this study, respectively. The %ξ values were calculated regarding the cLAS test value linked with PAV.SBSB because this bitumen exhibited the lower $\varphi^{p}$ among the studied bituminous materials. Hence, all %ξ values in Figure 7 are positive. “Supplementary Materials” include Figures S1–S4, which exhibit the DCCs of all types of bitumen associated with LASH test results of 25%, 50%, 75%, and 125% of $S_{f}$, respectively. In addition, each figure contains the DCCs related to 1, 5, 15, and 30 min of RPs, and these periods are named RP1, RP5, RP15, and RP30, respectively. Figures S1–S4 depict the DCCs up to the failure point defined by the SPC peak.

Figure 6 shows that %R_s_ generally increases and decreases while the RP and DL rise, respectively, regardless of the asphalt binder (in the pre-failure stage: 25%, 50%, and 75% of $S_{f}$). At the post-failure stage (125% of $S_{f}$), the %R_s_ values suddenly decline, and fail to follow the previously mentioned tendency linked with the RP. The main reason for this phenomenon is that all types of bitumen have surpassed the failure point. These findings agree with previous research works [24,25]. According to these results, the PSE peak seems to be a convenient failure definition to evaluate bitumen fatigue performance.

After analyzing Figure 6, it is possible to confirm that PAV.IPAB1.5 and PAV.NA exhibit eight and five times higher %R_s_ than the other types of bitumen in this study, respectively. Accordingly, these two materials generally exhibit superior self-restoration activity after the RP than the other asphalt binders. However, after evaluating the bitumen fatigue performance in Figures S1–S4, in terms of a DCC analysis, this research team realizes that PAV.IPAB0.5, PAV.STPB1.0, and PAV.STPB0.5 commonly exhibit the three higher responses (in this order). As a result, bitumen with a larger %R_s_ (PAV.IPAB1.5 and PAV.NA) fail to show superior fatigue performance in terms of a DCC assessment. This finding agrees with the previous study by Lv et al. [29]. Thus, this scenario confirms that the current framework fails to ensure a higher fatigue performance in terms of a DCC analysis for bitumen with a higher %R_s_ when assessing the fatigue behavior of a group of asphalt binders. This phenomenon justified the proposal of %ξ by Lv et al. [29].

Figure 7 illustrates the %ξ values associated with all types of bitumen in this study. This figure proves that %ξ generally increases when the RP and DL increase, regardless of the bitumen (in the pre-failure stage: 25%, 50%, and 75% of $S_{f}$). This finding related to the RP agrees with previous studies; for instance, Almutairi and Baaj [28]. Nevertheless, in this case, the finding linked with the DL is in the opposite direction of previous research works; for example, see Xie et al. [23] and Wang et al. [9], where these research teams reported lower performance while increasing the DL. The finding from Figure 7 proves that the combination effect of RPs, DLs, and adding a polymer into NA can promote or diminish the fatigue performance and self-restoration activity of modified bitumen, depending on the specific case. In addition, PAV.IPAB0.5 generally exhibits superior %ξ values than the other types of bitumen, regardless of the RP and DL. After analyzing all data linked with Figure 7, this research team concludes that STPU and IPA1w cause their highest values of %ξ at 1.0% (%ξ = 207.54) and 0.5% (%ξ = 232.64) contents concerning NA, respectively. Both results are obtained at DL = 75% and RP = 30 min, and these results confirm the finding from Figure 7 linked with the RP and DL.

The possible explanation for the finding related to the DL might be because the procedure foundations for calculating %R_s_ and %ξ are different. The %R_s_ reflects the material response (self-restoration) at one specific point on the DCC. By contrast, %ξ represents the bitumen behavior (self-restoration and fatigue performance) considering the whole extension of the DCC up to the point of interest. As a result, %ξ includes the influence of self-restoration activity on the asphalt binder fatigue response. The finding associated with %ξ values and Figure 7 could define a turning point and create a new foundation for studying bitumen fatigue performance. To date, researchers have believed and reported that a higher DL (up to the failure point) reduces the bituminous material fatigue behavior and the chance for self-restoration activity; for instance Xie et al. [23] and Wang et al. [9]. This research team believes that the possible explanation could be linked with the thixotropy phenomenon, dissipative energy, temperature, and viscosity (see deeper explanation in the Section 3). The finding linked with the DL and Figure 7 agrees with Lv et al.’s study [29].

Table 6 shows the fatigue performance rankings of all asphalt binders in all test conditions, linked with %ξ values. PAV.IPAB0.5, PAV.STPB1.0, and PAV.STPB0.5 exhibit the ranking numbers 1, 2, and 3, respectively; which means that, considering the effect of adding polymers into NA, RPs, and DLs, these asphalt binders show the three best fatigue performances in terms of %ξ analysis. Even though PAV.IPAB0.5 shows the ranking number 1 in the final ranking, in the case of the DL (25%$S_{f}$) and RP1, this bitumen reaches ranking number 2. The same phenomenon occurs with PAV.STPB1.0 and PAV.STPB0.5, because these asphalt binders do not always exhibit the ranking numbers 2 and 3, respectively, in Table 6, while changing the DLs and RPs. This fact demonstrates the influence of these parameters on the bitumen fatigue response. However, IPA1w promotes the highest %ξ value in NA (%ξ = 232.64), STPU shows more stability to improve the NA fatigue response because the sum of rankings (44 + 35 + 79 = 158) of the latter polymer is lower than that (17 + 67 + 112 = 196) of the former one.

When comparing each ranking performance linked with %ξ present in Table 6 with the corresponding DCC in Figures S1–S4, it is possible to realize that the ranking order of %ξ values and the corresponding DCCs match. This fact confirms that the newly proposed composite procedure to evaluate the self-restoration activity and fatigue performance of the bituminous materials exhibits the power to ensure a higher fatigue performance in terms of a DCC analysis for bitumen with a higher self-restoration activity when assessing the fatigue behavior of a group of asphalt binders (capacity 1). Furthermore, it ensures the ranking consistency between bitumen failure definition and its fatigue performance in terms of a DCC analysis when appraising the fatigue behavior of numerous asphalt binders (capacity 3).

When comparing the error bars present in Figure 6 and Figure 7, it is possible to conclude that the error bars linked with the former figure are larger than those related to the latter figure. This scenario demonstrates that the proposed composite procedure shows superior accuracy to evaluate bitumen self-restoration activity and fatigue response than the current framework, because the calculated parameter values from the new proposal process change in a smaller range than those related to the current framework. This fact confirms the superiority of the new proposed methodology with respect to the current framework.

### 3.2. Analysis of %β (Respect to cLAS of PAV.SBSB) Values

**Figure 8:**
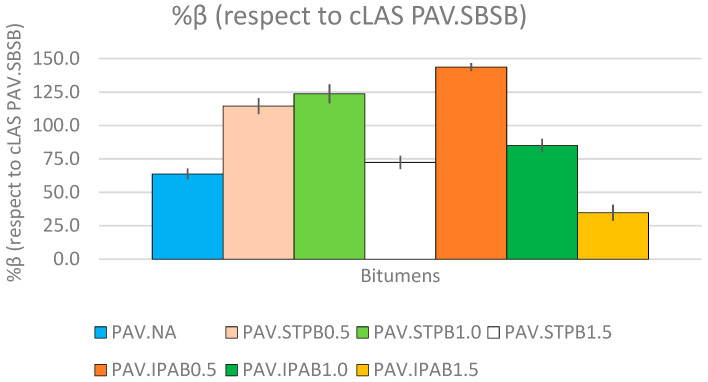
%β values of asphalt binders with respect to cLAS of PAV.SBSB.

Figure 8 depicts %β values related to PAV.NA, PAV.STPB0.5, PAV.STPB1.0, PAV.STPB1.5, PAV.IPAB0.5, PAV.IPAB1.0, PAV.IPAB1.5 regarding PAV.SBSB. The horizontal axis that intercepts the vertical axis at %β equal to 0 represents the %β value associated with PAV.SBSB. Figure S5 (Supplementary Materials) shows the DCCs linked with all asphalt binders obtained from the cLAS test results. These DCCs are plotted up to the $S_{f}$ related to each asphalt binder. Table 7 displays the fatigue behavior ranking of bitumen related to the %β parameter.

Figure 8 shows that when the STPU content in NA increases, the fatigue performance (%β value) of STPB first increases and then decreases. However, while increasing the IPA1w content in NA, the fatigue behavior (%β value) of IPAB always decreases. PAV.STPB1.0 exhibits a higher %β value (123.7%) than the other two STPB and PAV.IPAB0.5 shows a superior %β value (143.7%) than those linked with PAV.IPAB1.0 and PAV.IPAB1.5. Hence, 1.0% of STPU and 0.5% of IPA1w contents are the most convenient amount of these self-healing polymers to mix with NA. Nevertheless, the fatigue performance (%β value) of IPAB always decreases while increasing the IPA1w content in NA; PAV.IPAB0.5 displays the highest %β value among the tested bitumen in this study. In addition, PAV.IPAB1.5 shows the lowest %β value among the SPB in this research work and is even lower than that linked with PAV.NA. Figure 8 demonstrates that these room-temperature self-healing polymers can promote the fatigue response of SPB, by mixing the convenient proportion of this type of polymer and NA.

In Figure S5, the DCCs linked with PAV.IPAB0.5, PAV.STPB1.0, and PAV.STPB0.5 exhibit superior fatigue performance than the other types of bitumen in this research work. The DCCs of these bituminous materials almost overlap in the *C* vs. *S* graph, which means that the *C*–*S* relationships linked with PAV.IPAB0.5, PAV.STPB1.0, and PAV.STPB0.5 are almost the same. However, these asphalt binders reach the failure point at different positions in the abovementioned graph, where the PAV.IPAB0.5 even reaches the $S_{f}$ at a lower *C* value than those linked with PAV.STPB1.0 and PAV.STPB0.5 and exhibits superior fatigue performance than these two materials because it reaches the failure point at a higher *S* value. This conclusion agrees with Figure 8. This fact demonstrates the importance of *C*–*S* relationships and the area below the DCC to assess the bitumen fatigue performance. These two parameters are included when calculating %β values; Table 7 displays their rankings. This table agrees with Figure 8 regarding the fatigue response. It demonstrates that although IPA1w promotes the highest %β in this study, STPU shows more stability to promote NA fatigue performance (without considering the RP and DL), because the sum of rankings (equal to 10) linked with STPB is lower than that associated with IPAB (sum equal to 12).

When comparing the rankings associated with the %β values in Table 7 and the corresponding DCCs in Figure S5, this research team confirms that the ranking order of %β values and the corresponding DCCs match. Furthermore, Table 7 confirms that bitumen with a higher fatigue failure definition show a greater fatigue performance in terms of a DCC analysis. Hence, this fact proves that the proposed procedure ensures a higher fatigue performance in terms of a DCC analysis for bitumen with a higher failure definition when analyzing the fatigue response of various bituminous materials (capacity 2). Moreover, it ensures ranking consistency between the bitumen failure definition and its fatigue performance in terms of a DCC analysis when appraising the fatigue behavior of numerous asphalt binders (capacity 3).

### 3.3. Analysis of %δ (Respect to cLAS of PAV.SBSB) Values

**Figure 9:**
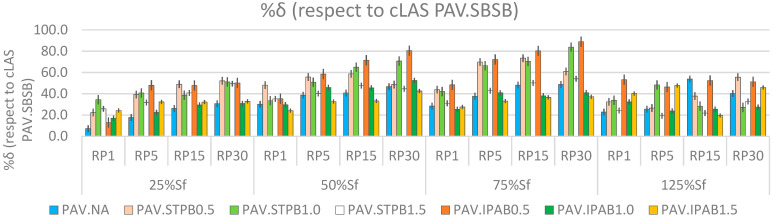
%δ values of asphalt binders with respect to cLAS of PAV.SBSB.

Figure 9 depicts %δ values of all types of bitumen at different DLs and RPs concerning cLAS related to PAV.SBSB. Table 8 displays the fatigue performance rankings of all asphalt binders at different test conditions associated with %δ values.

Figure 9 shows that %δ values commonly grow when the RP and DL increase at the pre-failure stage (DL = 25%, 50%, or 75% of $S_{f}$), regardless of the asphalt binder. The finding associated with the RP agrees with previous research works; see Almutairi and Baaj [28]. Nevertheless, in this case, the finding related to DL fails to support the conclusions from previous studies; for instance, Xie et al. [23] and Wang et al. [9], because these research works found lower self-restoration activity and fatigue response when increasing the DL. The findings from Figure 7 and Figure 9 agree, which confirms the consistency of the conclusion. At the post-failure stage (DL = 125% of $S_{f}$), %δ values commonly decrease concerning those values related to 75% of $S_{f}$, regardless of the bitumen. This fact confirms the failure occurrence and demonstrates the efficacy of the SPC peak as a failure definition.

In addition, STPB and IPAB generally exhibit higher %δ values than those associated with NA. This phenomenon demonstrates the efficiency of the two room-temperature self-healing polymers to promote the self-restoration activity in NA when applying RPs at different DLs. As a result, this specific type of polymer could soon become a common asphalt binder modifier to incentive the self-restoration action on asphalt pavement surface and layers. Hence, the service life of asphalt pavement could be extended.

The finding from Figure 9 demonstrates that the combination of RP and DL effects promotes the self-restoration of asphalt binders, regardless of the material. This finding represents a turning point because, until now, research works have found that applying the RP at a higher DL diminishes the bitumen fatigue response, as mentioned before. Self-restoration mainly includes self-healing and thixotropy, as commented before. The former represents the reduction of fracture cracks and expanding the effective area, which is the area without cracks [48]. The latter signifies a diminished dynamic shear modulus at the loading cycle stage due to the breakdown of the microstructure and restoration of the dynamic shear modulus when removing the loading cycle due to the building-up of the microstructure [48]. Hence, this research team believes that the combination of both these phenomena produce an internal process in the asphalt binders that allows them to promote self-restoration activity. This internal process is explained in detail in the Section 3.

In Table 8, PAV.IPAV0.5, PAV.STPB0.5, and PAV.STPB1.0 exhibit the ranking numbers 1, 2, and 3 (see “Final ranking”), respectively, in terms of %δ value analysis. This finding means that these three types of bitumen show the three higher self-restoration rates when the combination effect of the RP and DL is simultaneously considered. PAV.IPAV0.5 does not always show the ranking (number 1), as mentioned above, when changing the DL (from 25% to 125% of $S_{f}$) at different RPs. This phenomenon also occurs with PAV.STPB0.5 and PAV.STPB1.0, because the former and the latter do not always exhibit ranking 2 and 3, respectively. This fact means that the RP and DL influence bitumen self-restoration activity. Moreover, STPU exhibits more stability in promoting the self-restoration in NA than IPA1w, because the sum of the rankings linked with STPB (sum = 9) is lower than that value corresponding to IPAB (sum = 12).

Table 9 shows the final rankings associated with %ξ, %β, and %δ. After comparing the final rankings related to %ξ (considering the effect of adding a polymer into NA, RP, and DL) and %β (only considering the effect of adding a polymer into NA), it is possible to conclude that the RP and DL do not have a critical influence in the results because, when removing these parameters, the final rankings are the same. However, the corresponding results change when comparing final rankings related to %ξ and %δ (considering the effect of the RP and DL). Hence, self-healing polymers significantly influence the bitumen fatigue response and self-restoration action because the results change when its effect is removed. This fact confirms that mixing the convenient polymer with NA (with the proper content) makes it possible to promote the self-restoration activity in the base asphalt binder to increase its fatigue performance.

### 3.4. Discussion

This research team considers that the %R_s_ proposed by Aurilio et al. [27] has a limited view on asphalt binder fatigue performance because it only evaluates the bitumen behavior at one specific point of the DCC (during RP). As a result, this parameter cannot assess the performance of the bituminous materials before and after the RP. However, these two phases of the LASH test have a decisive influence on the bitumen’s final response. For instance, Xie et al. [23] reported that in some cases, the DCCs linked with the LASH test, after the RP, collapsed with respect to the corresponding DCC related to the cLAS test. However, %R_s_ fails to evaluate the occurrence of this phenomenon because it just analyzes the behavior of the asphalt binder during the rest period, without considering the bitumen response after the RP. This fact confirms that this parameter exhibits a limited power to assess the bitumen fatigue performance.

“Supplementary Materials” include Figures S6–S13. These figures represent the DCCs of LASH at different RPs and DLs linked with PAV.NA, PAV.STPB0.5, PAV.STPB1.0, PAV.STPB1.5, PAV.IPAB0.5, PAV.IPAB1.0, PAV.IPAB1.5, and PAV.SBSB, respectively. These figures comprise the DCCs associated with the cLAS test as a reference curve. This study also reports in the “Supplementary Materials” some DCCs (linked with the LASH test) that slightly collapse concerning the corresponding DCC (associated with cLAS). The cases are as follows: Figure S6 (a) PAV.NA (RP1 and 25%$S_{f}$), PAV.NA (RP1 and 125%$S_{f}$), (b) PAV.NA (RP5 and 25%$S_{f}$), PAV.NA (RP5 and 125%$S_{f}$); Figure S7 (b) PAV.STPB0.5 (RP5 and 125%$S_{f}$), (c) PAV.STPB0.5 (RP15 and 125%$S_{f}$); Figure S8 (a) PAV.STPB1.0 (RP1 and 25%$S_{f}$), PAV.STPB1.0 (RP1 and 125%$S_{f}$), (b) PAV.STPB1.0 (RP5 and 125%$S_{f}$), (c) PAV.STPB1.0 (RP15 and 125%$S_{f}$), (d) PAV.STPB1.0 (RP15 and 125%$S_{f}$); Figure S9 (a) PAV.STPB1.5 (RP1 and 125%$S_{f}$), (b) PAV.STPB1.5 (RP5 and 125%$S_{f}$), (c) PAV.STPB1.5 (RP15 and 125%$S_{f}$); Figure S10 (a) PAV.IPAB0.5 (RP1 and 25%$S_{f}$) and PAV.IPAB0.5 (RP1 and 50%$S_{f}$); Figure S11 (a) PAV.IPAB1.0 (RP1 and 25%$S_{f}$), (b) PAV.IPAB1.0 (RP5 and 25%$S_{f}$), PAV.IPAB1.0 (RP5 and 125%$S_{f}$), (d) PAV.IPAB1.0 (RP30 and 75%$S_{f}$); Figure S12 (c) PAV.IPAB1.5 (RP15 and 125%$S_{f}$).

Hence, when conducting the LASH test at different RPs and DLs, the tendency of %R_s_ values fails to reflect the actual fatigue behavior of asphalt binders during this test. This fact explains why bitumen with higher %R_s_ in Figure 6 differ from asphalt binders with superior fatigue performance in terms of a DCC analysis, in Figures S1–S4. As a result, the %R_s_ parameter is no longer convenient to evaluate bitumen performance. In contrast, the %ξ can evaluate the bituminous material response before, during, and after the RP when carrying out the LASH test at diverse RPs and DLs because this parameter evaluates the area below the DCC up to the point of interest. As %ξ assesses the influence of adding a polymer into NA, RPs, and DLs on bitumen fatigue response, simultaneously, this research work introduced the following parameters: %β and %δ. The former parameter only evaluates the effect of mixing a polymer and NA on the fatigue performance of the base asphalt binder. The latter appraises the influence of DLs and RPs on bitumen fatigue response, as explained before. This research team wants to clarify that the %ξ, %β, and %δ parameters represent an integral view of the DCC that shows the total (from the starting point of the LASH test to the point of interest) and actual fatigue performance of the testing asphalt binders. Depending on which factors (adding a polymer into NA, RPs, and DLs) the researchers want to evaluate, it is possible to use any of these parameters.

In the Section 3.1, Figure 7 shows that %ξ generally increases when the RP and DL increase. This fact means the bituminous materials in this study commonly improve their fatigue performance, considering all factors (addition of a polymer into NA, RP, DL), while reaching closer to the failure point (in the pre-failure stage). This phenomenon represents a breakthrough finding because, until now, research works have found that applying the RP at a higher DL (before the failure point) reduces the bitumen fatigue response. For instance, Wang et al. [49] and Wang et al. [50], studying and fully understanding the self-restoration phenomenon in the bituminous materials is crucial to fully understanding and modeling the fatigue response of asphalt pavement materials [49]. As a result, the explanation of the possible reason for the phenomenon found in Figure 7 is as follows:

The dissipative viscoelastic energy activity of bitumen can cause a temperature increment of this material while being subjected to loading cycles (cLAS or LASH test). This process causes the internal temperature in asphalt binder specimens to rise over the actual existing ambient temperature. Then, the asphalt binder cools back to its original temperature when the load is removed [51]. The temperature change causes the variation of dynamic shear modulus [52]. The phenomenon when the bitumen cools back to the ambient temperature is named “self-cooling” [53]. Thixotropy is an asphalt binder intrinsic property that diminishes its dynamic shear modulus during the loading phase because of microstructure breakdown and recovers its dynamic shear modulus when eliminating the load because of the building-up of the microstructure [52]. The evolution of asphalt material thixotropy highly depends on temperature augmentation. As a result, bitumen viscosity also influences this phenomenon [54]. Moreover, the temperature increment promotes the healing activity [55], and this phenomenon represents the fracture crack reduction and effective area growth (which is an uncracked area) [52].

As a result, this research team believes that by applying the RP at a higher DL (before the failure point), the asphalt binder will reach a higher internal temperature because of these phenomena mentioned above when applying loading cycles. If the asphalt binder reaches a superior internal temperature, its dynamic shear modulus and viscosity decrease. According to the explanation, this scenario creates the condition to promote thixotropy and self-healing processes during the RP. Hence, the material will have a superior opportunity to recover a certain percent of its original stage, dynamic shear modulus, and strength capacity, even though the bituminous material has a higher DL. This research team believes the asphalt binder follows the process described above while conducting LASH and applying the RP before the failure point. Because the *S* values are not high enough to prevent the bitumen from recovering a certain percentage of their original stage.

To prove the previous possible explanation (theory) of the finding from Figure 7, this research team will utilize the following equations: dissipated energy (Equation (18)), cumulative dissipated energy (Equation (19)), and heat energy (Equation (20)).

(18) $$W_{n}=\pi \cdot \tau_{n}\cdot \gamma_{n}\cdot sin\delta_{n}$$

(19) $$CDE=\sum_{i=1}^{n}W_{i}$$

(20) Q=m·c·∆T

**Figure 10:**
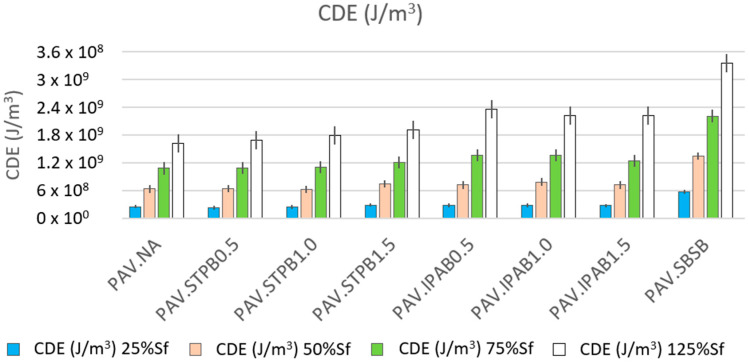
Obtained CDE values of all asphalt binders up to 25%, 50%, 75%, and 125% of $S_{f}$.

In Equation (18), $W_{i}$, $\tau_{i}$, $\gamma_{i}$, and $\delta_{i}$ represent the dissipated energy at the *i*-th loading cycle, shear stress, shear strain, and phase angle at the *i*-th loading cycle, respectively. In Equation (19), the CDE illustrates the cumulative dissipated energy which is the sum of all dissipated energies up to the cycle of interest (cycle n) [6,56]. In Equation (20), Q, m, c, and ∆T show the heat energy, mass, specific heat capacity, and change in temperature, respectively [57]. The $W_{i}$ and CDE related to each type of bitumen in this study can be determined using the LAS and LASH test results. Figure 10 shows the obtained CDE of all asphalt binders up to 25%, 50%, 75%, and 125% of $S_{f}$, which means this figure illustrates the reached CDE values when the RP starts while carrying out the LASH test. As a result, these CDE values are the amounts of dissipated energy when the bitumen self-restoration process starts, and each CDE value is the average of the CDE amounts associated with the four LASH test results in each DL (because of four different RPs).

**Figure 11:**
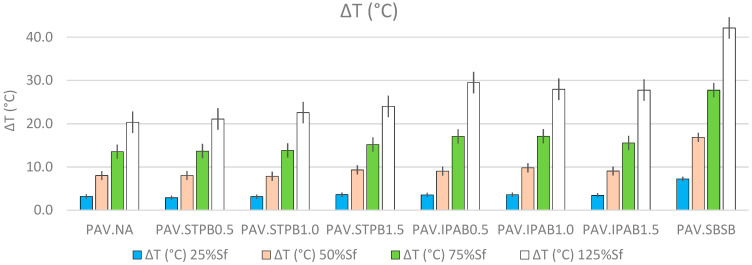
Obtained ∆T values of all asphalt binders up to 25%, 50%, 75%, and 125% of $S_{f}$.

Figure 10 proves that while increasing the DL at which the RP is applied, the CDE values increase, regardless of the asphalt binders. CDE rises because of the cyclic loading input on the asphalt binder samples. This fact confirms the above possible explanation for the finding in Figure 7. In addition, PAV.SBSB shows higher CDE values than the other types of bitumen, regardless of the DL. Despite this finding from Figure 10, this research team considers it necessary to analyze the ∆T and viscosity values. The ∆T parameter was calculated by transforming Equation (20), utilizing the calculated CDE values and the volume of the asphalt binder samples. Figure 11 shows the determined ∆T values of all asphalt binders up to 25%, 50%, 75%, and 125% of $S_{f}$. Hence, this figure exhibits the reached ∆T values when the RP starts during the LASH test. As a result, these ∆T values are the amounts of temperature variation when the bitumen self-restoration process starts, and each ∆T value is the average of the ∆T amounts linked with the four LASH test data in each DL (because of four different RPs).

**Figure 12:**
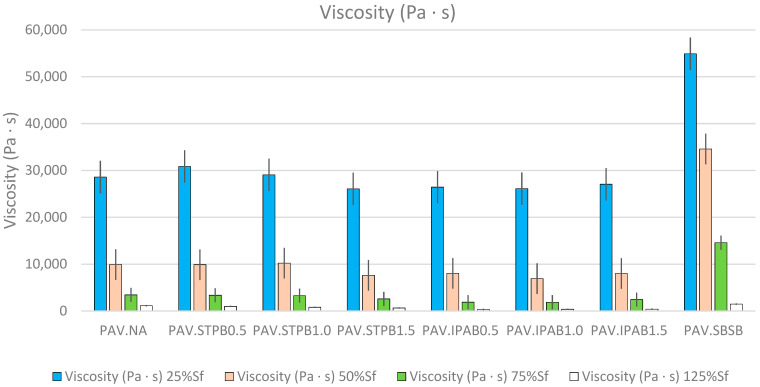
The viscosities of all types of bitumen up to 25%, 50%, 75%, and 125% of $S_{f}$.

Figure 11 demonstrates that ∆T increases while increasing the DL at which the RP is applied, regardless of asphalt binders, when conducting the LASH test. This finding from Figure 11 agrees with the previous possible explanation of the finding from Figure 7. It is interesting to highlight that PAV.SBSB shows a higher ∆T than the other types of bitumen in this study, but this asphalt binder failed to exhibit higher self-restoration in the Section 3. This fact demonstrates that even though temperature influences bitumen self-restoration activity, other factors also affect this parameter, such as the viscosity, as mentioned before. Figure 12 depicts the viscosities of all types of bitumen up to 25%, 50%, 75%, and 125% of $S_{f}$. Hence, this figure illustrates the reached viscosity values when the RP starts while conducting the LASH test. Hence, these viscosities are the bitumen consistencies when its self-restoration activity starts, and each viscosity is the average of those values associated with the four LASH test data in each DL (because of the four different RPs). The viscosities were calculated considering the ∆Ts, the set temperature in LASH (28 °C), and the power equations obtained from the known viscosities at various specific temperatures.

Figure 12 reveals that viscosity decreases while increasing the DL at which the RP is applied, regardless of the asphalt binder, when undertaking the LASH test. This finding proves the influence of the temperature increment in this parameter. Although PAV.SBSB exhibits a higher ∆T than the other asphalt binders in Figure 11, this bitumen shows higher viscosity than the other asphalt materials in Figure 12. The reason for this finding is that PAV.SBSB has a higher temperature resistance than the other bituminous materials in this study. The softening point parameter can explain this phenomenon; the value related to SBSB is 83.2 °C, while that linked with NA and the SPB is 51.1. As a result, SBSB needs more energy (temperature) to reduce its consistency. Hence, PAV.SBSB exhibits higher viscosity than the other asphalt binders in this research work, which explains why this bitumen exhibited in Section 3 a poor self-restoration activity.

The findings from Figure 10, Figure 11 and Figure 12 provide the reasons to explain why the %ξ increment occurs while the DL increases (before the failure point) to a certain extent. Furthermore, these figures confirm the influence of CDE, ∆T, and viscosity on bitumen self-restoration capacity, and the combination of their effects can promote or demote the fatigue response of asphalt binders. However, more experiments are needed to understand this phenomenon fully. Moreover, this research team recommends analyzing the whole DCC (up to the point of interest) instead of one specific point to assess the bitumen fatigue performance precisely. The finding from Figure 7 agrees with the study by Lv et al. [29].

The ranking analysis of the bitumen performance is also essential to decide which material should be used depending on the project characteristics. Table 9 illustrates the final rankings after evaluating the results related to %ξ, %β, and %δ. When comparing the %β final rankings with those of %ξ (removing the influence of RPs and DLs on the bitumen fatigue response), this research team confirms that the final rankings are the same. This finding does not mean that the RP and DL do not influence asphalt binder performance because, when these parameters change in Table 6, the ranking positions of the asphalt binders change. Hence, both the RP and DL influence bitumen response, but their effects are not high enough to change the final rankings, according to the %β and %ξ assessment. In addition, the final rankings differ when comparing %δ results regarding those of %ξ (deleting the effect of adding polymers). This finding proves that the influence of adding polymers into NA is high enough to change the final rankings, according to the %δ and %ξ data evaluation, as mentioned before.

Consistent with the findings in the previous paragraph, this research team can confirm that adding a polymer into NA has more influence on bitumen fatigue performance than the RP and DL (*S* value), according to the %ξ, %β, and %δ evaluation, even though previous research works have highlighted the importance of these two parameters (RP and DL). For instance, Pérez-Jiménez et al. [58] confirmed the strong influence of RP on the fatigue performance of asphalt binders; and Motamedi et al. [59] reported that *S* values have a considerable effect on bitumen fatigue life.

In Section 3.1 of this study, the newly proposed framework exhibited the capacity to ensure a higher fatigue performance in terms of a DCC analysis for bitumen with a higher self-restoration activity when assessing the fatigue behavior of a group of asphalt binders. In addition, it ensured ranking consistency between bitumen failure definition and its fatigue performance in terms of a DCC analysis when appraising the fatigue behavior of numerous asphalt binders. In Section 3.2 of this research work, the proposed procedure guaranteed a higher fatigue performance in terms of a DCC analysis for bitumen with a higher failure definition when analyzing the fatigue response of various bituminous materials. Moreover, it assured a ranking consistency between bitumen failure definition and its fatigue performance in terms of a DCC analysis when appraising the fatigue behavior of numerous asphalt binders. As a result, the newly proposed procedure could match the three capabilities mentioned in the Section 1 simultaneously. Hence, the newly proposed process is the only one that can match those three requirements at the same time.

Table 10 illustrates the capacities (advantages) to be ensured by a framework to evaluate the bitumen fatigue response and its self-restoration activity successfully. This table illustrates which capabilities can be ensured by the current, previously proposed, and newly proposed frameworks.

Furthermore, the Glover–Rowe parameter mainly depends on G* and phase angle (δ) to assess the fatigue cracking performance of asphalt binders [60]. However, Safaei et al. [61] proved that δ failed to be an appropriate parameter to appraise the bitumen fatigue behavior because its tendency was unclear. However, researchers worldwide have used this parameter (δ) to evaluate the fatigue performance of asphalt mixtures for many years. Hence, the results and conclusions from the Glover–Rowe parameter are not trustworthy. This fact represents the main disadvantage of the Glover–Rowe parameter concerning the proposed composite procedure in this study. Because the proposal does not include δ in any formula. In addition, the proposal in this research work can be used to evaluate the self-restoration capacity of bitumen, while the Glover–Rowe parameter failed to have this capacity.

Future research works of this team will focus on a better understanding of the fatigue phenomenon related to the %ξ, %β, and %δ parameters. Future studies will also include the design of asphalt mixtures with SPB and assess the relationship between fatigue performance and the microstructure of asphalt binders. Moreover, the findings related to the %ξ, %β, and %δ parameters can change the factors to consider the convenient bitumen for one specific project and the criteria for evaluating the bitumen fatigue response.

## 4. Conclusions

This study proposed a new procedure to analyze the self-restoration capacity and fatigue performance of asphalt binders simultaneously utilizing LAS, LASH, and S-VECD methods. This proposal combined a new failure definition based on *S* values instead of N values and a new procedure to evaluate the bitumen fatigue response and self-restoration capacity (all together) based on the area below the DCCs. This research work introduced two new parameters to assess the asphalt binder’s response better. This study analyzed eight different bituminous materials to verify the efficiency of the new proposal. After analyzing all experimental results, the following conclusions can be drawn:

- The newly proposed procedure was able to simultaneously match the following three capacities: to ensure a higher fatigue performance in terms of a DCC analysis for bitumen with a higher self-restoration activity; to ensure a higher failure definition when assessing the fatigue behavior of a group of asphalt binders; and to ensure ranking consistency between bitumen failure definition and its fatigue performance in terms of a DCC analysis when appraising the fatigue behavior of numerous asphalt binders.
- Adding a room-temperature self-healing polymer into NA had a higher effect on bitumen fatigue performance and self-restoration activity than the combined effect of both the RP and DL.
- Both room-temperature self-healing polymers (STPU and IPA1w) successfully increased the fatigue response and self-restoration capacity of asphalt binders.
- Bitumen fatigue performance increased while increasing the *S* value at which the RP was applied (in the pre-failure stage) because the longer the loading cycle, the higher the cumulative dissipative energy, temperature variation, and reduction of viscosity. This scenario created the conditions for superior self-restoration activity.
- STPB and IPAB showed their highest %ξ values, containing 1.0% of STPU (%ξ = 207.54) and 0.5% of IPA1w (%ξ = 232.64), respectively. In both cases, the results were obtained at DL = 75% of $S_{f}$ and RP = 30 min.
- The %R_s_ parameter failed to assess the actual fatigue response of asphalt binder effectively.
- The newly proposed procedure had the following capacities: to evaluate the effect of adding a polymer into NA on the bitumen fatigue performance and self-restoration activity; to assess the combined influence of the DL and RP on the bitumen fatigue response and self-restoration action; and simultaneously to appraise the combined effect of adding a polymer into NA, DL, and RP on the fatigue behavior of bituminous material.

## Figures and Tables

**Table 1:** Summary of main studies linked with bitumen self-healing performance and issues.

**Study**	**Bitumen**	**Framework**	Conclusions	Failure Definition	Issues
Xie et al. [23]	NA and SBSB	LAS, LASH, and S-VECD	%Hs (NA) > %Hs (SBSB)	Stored PSE peak to define $N_{f}$.	Current framework fails to ensure superior fatigue performance in terms of DCC for bitumen with higher $N_{f}$ and %Hs. Current framework exhibits ranking inconsistency between $N_{f}$ and bitumen fatigue performance in terms of DCC.
Wang et al. [24]	NA and SBSB	LAS, LASH, and VECD	Aging process and SBS decreased %Hs. Light fractions, small molecules, and longer molecules increased %Hs	Stored PSE peak to define $N_{f}$.	Current framework fails to ensure superior fatigue performance in terms of DCC for bitumen with higher $N_{f}$ and %Hs. Current framework exhibits ranking inconsistency between $N_{f}$ and bitumen fatigue performance in terms of DCC.
Wang et al. [9]	NA and SBSB	LAS, LASH, and VECD	Superior number of saturates/aromatic fractions and small molecules promoted %Hs. %Hs (NA) ≈ %Hs (SBSB)	Stored PSE peak to define $N_{f}$.	Current framework fails to ensure superior fatigue performance in terms of DCC for bitumen with higher $N_{f}$and %Hs. Current framework exhibits ranking inconsistency between $N_{f}$ and bitumen fatigue performance in terms of DCC.
Aurilio [25] and Aurilio and Baaj [26]	SPB and SBSB	LAS, LASH, simplified LASH, and S-VECD	Self-healing polymer promoted elastomeric properties but could not promoted %Hs. SBS promoted %Hs.	Stored PSE peak to define $N_{f}$.	Current framework fails to ensure superior fatigue performance in terms of DCC for bitumen with higher $N_{f}$ and %Hs. Current framework exhibits ranking inconsistency between $N_{f}$ and bitumen fatigue performance in terms of DCC.
Aurilio et al. [27]	WMAB and NA	LAS, simplified LASH, and S-VECD	%Hs (aged NA) > %Hs (aged WMAB)	Stored PSE peak to define $N_{f}$.	Current framework fails to ensure superior fatigue performance in terms of DCC for bitumen with higher $N_{f}$ and %Hs. Current framework exhibits ranking inconsistency between $N_{f}$ and bitumen fatigue performance in terms of DCC.
Almutairi and Baaj [28]	NA, SBSB, GPB, GPCMB	LAS, LASH and S-VECD	GPCM maintained and improved %Hs. Glass powder increased %Hs.	Stored PSE peak to define $N_{f}$.	Current framework fails to ensure superior fatigue performance in terms of DCC for bitumen with higher $N_{f}$ and %Hs. Current framework exhibits ranking inconsistency between $N_{f}$ and bitumen fatigue performance in terms of DCC.
Lv et al. [29]	NA and STPB	LAS, LASH and S-VECD	$\mathrm{Higher}\;N_{f}$ or %Hs could not ensure higher fatigue performance in DCC. Ranking inconsistency between $N_{f}$ and DCC.	Stored PSE peak to define $N_{f}$.	Proposal of new procedure to ensure higher fatigue performance in terms of DCC for bitumen with higher %Hs. Current framework fails to ensure superior fatigue performance in terms of DCC for bitumen with higher $N_{f}$. Current framework exhibits ranking inconsistency between $N_{f}$ and bitumen fatigue performance in terms of DCC.
Lv et al. [30]	NA, STPB, IPAB, and SBSB	LAS and S-VECD	$\mathrm{Higher}\;N_{f}$ could not ensure higher fatigue performance in DCC. Ranking inconsistency between $N_{f}$ and DCC.	SPC peak (see Equation (8) and Section 2.10 in general) to define $S_{f}$.	Proposal of new framework to ensure higher fatigue performance in terms of DCC for bitumen with higher failure definition (based on S) and eliminate the ranking inconsistency between failure definition (based on S) and bitumen fatigue performance in terms of DCC. Proposed framework fails to ensure superior fatigue performance in terms of DCC for bitumen with higher %Hs.

**Table 2:** Physical properties of NA.

Tests	Standard Value	Measured Value	Standard Test
Penetration (25 °C, 5 s, 100 g) (0.1 mm)	60~80	60.1	T0604
Penetration index (PI)	−1.5~1.0	−0.4	T0604
Softening point (°C)	≥46	51.1	T0606
Viscosity (60 °C) (Pa · s)	≥180	219	T0620
Ductility (10 °C)	≥45	62	T0605
Wax content (%)	≤2.2	1.8	T0615
Flash point (°C)	≥260	300	T0611
Density (15 °C) (g/cm^3^)	-	1.033	T0603
Solubility (%)	≥99.5	99.91	T0607
After RTFO ^1^: Mass change (%) Residual penetration ratio (%) Residual ductility (10 °C)	≤±0.8 ≥61 ≥0.6	0.021 67 8	T0609 T0604 T0605

^1^ Rolling thin film oven (RTFO) test.

**Table 3:** Physical properties of SBSB.

Tests	Standard Value	Measured Value	Standard Test
Penetration (25 °C, 5 s, 100 g) (0.1 mm)	30~60	52.0	T0604
Penetration index (PI)	≥0	0.15	T0604
Softening point (°C)	≥76	83.2	T0606
Viscosity (135 °C) (Pa · s)	≤3	2.45	T0625
Ductility (5 °C)(cm)	≥25	35	T0605
Flash point (°C)	≥230	310	T0611
Solubility (%)	≥99.0	99.78	T0607
SBS block ratio (B/S)	-	70/30	-
SBS molecular weight (g/mol)	-	120,000	-
SBS content (%)	-	5	-
After RTFO ^1^: Mass change (%) Residual penetration ratio (%) Residual ductility (10 °C) (cm)	≤±1.0 ≥65 ≥20	−0.04 78 22	T0610 T0604 T0605

^1^ the three tests in this section were conducted after the RTFO test.

**Table 4:** Physical properties of STPU.

Parameters	STPU Values
Tensile strength (MPa)	13.5 ± 2.2
Elongation (dried state, %)	1460 ± 87
Density (g/cm^3^)	1.07
Melting point (°C)	120 ^a^
Molecular weight (g/mol)	72,700

^a^ = obtained from the temperature sweeping of the rheological test.

**Table 5:** Physical properties of IPA1w.

Parameters	IPA1w Values
Tensile strength (MPa)	1.61 ± 0.15
Elongation (dried state, %)	1700
Young’s modulus (MPa)	0.59 ± 0.02
Toughness (MJ m^−3^)	17.89 ± 0.18
Molecular weight (g/mol)	82,000

**Table 6:** Bitumen fatigue performance rankings related to %ξ.

Bitumen (Aged-PAV)	Materials Ranking																Sum of Rankings	Final Rankings
	$25\%S_{f}$				$50\%S_{f}$				$75\%S_{f}$				$125\%S_{f}$					
	RP1	RP5	RP15	RP30	RP1	RP5	RP15	RP30	RP1	RP5	RP15	RP30	RP1	RP5	RP15	RP30		
NA	6	6	6	6	6	6	6	6	6	6	6	6	6	6	4	6	94	6
STPB0.5	3	3	2	3	2	3	3	3	3	3	3	3	3	3	2	2	44	3
STPB1.0	1	2	3	2	3	2	2	2	2	2	2	2	2	2	3	3	35	2
STPB1.5	5	5	5	4	5	5	5	5	5	5	5	4	5	5	6	5	79	5
IPAB0.5	2	1	1	1	1	1	1	1	1	1	1	1	1	1	1	1	17	1
IPAB1.0	4	4	4	5	4	4	4	4	4	4	4	5	4	4	5	4	67	4
IPAB1.5	7	7	7	7	7	7	7	7	7	7	7	7	7	7	7	7	112	7
SBSB	8	8	8	8	8	8	8	8	8	8	8	8	8	8	8	8	128	8

**Table 7:** Bitumen fatigue performance rankings related to %β.

Bitumen (Aged-PAV)	Rankings	$S_{f}$
NA	6	4.1093 × 10^9^
STPB0.5	3	5.4366 × 10^9^
STPB1.0	2	5.8173 × 10^9^
STPB1.5	5	4.6466 × 10^9^
IPAB0.5	1	6.7065 × 10^9^
IPAB1.0	4	5.0776 × 10^9^
IPAB1.5	7	3.7191 × 10^9^
SBSB	8	3.2469 × 10^9^

**Table 8:** Bitumen fatigue performance rankings related to %δ.

**Bitumen (Aged-PAV)**	Materials Ranking																Sum of Rankings	Final Rankings
	$25\%S_{f}$				$50\%S_{f}$				$75\%S_{f}$				$125\%S_{f}$					
	**RP1**	**RP5**	**RP15**	**RP30**	RP1	RP5	RP15	RP30	RP1	RP5	RP15	RP30	RP1	RP5	RP15	RP30		
NA	7	7	7	7	5	6	6	5	5	6	5	5	7	5	1	4	88	7
STPB0.5	4	3	1	1	1	2	3	4	2	2	2	3	4	4	3	1	40	2
STPB1.0	1	2	4	2	4	3	2	2	3	3	3	2	3	1	4	7	46	3
STPB1.5	2	5	3	4	3	5	4	6	4	4	4	4	6	7	6	5	72	4
IPAB0.5	6	1	2	3	2	1	1	1	1	1	1	1	1	3	2	2	29	1
IPAB1.0	5	6	6	6	6	4	5	3	7	5	6	6	5	6	5	6	87	6
IPAB1.5	3	4	5	5	7	7	7	7	6	7	7	7	2	2	7	3	86	5
SBSB																		

**Table 9:** Final rankings related to %ξ, %β, and %δ.

Bitumen (Aged-PAV)	Final Rankings (Table 6 [%ξ])	Final Rankings (Table 7 [%β])	Final Rankings (Table 8 [%δ])
NA	6	6	7
STPB0.5	3	3	2
STPB1.0	2	2	3
STPB1.5	5	5	4
IPAB0.5	1	1	1
IPAB1.0	4	4	6
IPAB1.5	7	7	5
SBSB	8	8	-

**Table 10:** Capacities of current, previously proposed, and newly proposed frameworks.

Capacities to be Ensured by a Framework	Current Framework	Framework from Lv et al. [29]	Framework from Lv et al. [30]	Newly Proposed Framework
Higher fatigue performance in terms of DCC assessment for bitumen with higher failure definition.	No	No	Yes	Yes
Higher fatigue performance in terms of DCC analysis for bitumen with higher self-restoration.	No	Yes	No	Yes
Ranking consistency between failure definition and fatigue performance in terms of DCC assessment.	No	No	Yes	Yes
Assessing bitumen self-restoration and fatigue performance simultaneously.	No	Yes	Yes	Yes
Differentiating the effect of adding polymer into NA on self-restoration and fatigue performance simultaneously from the effect of DL and RP.	No	No	No	Yes
Differentiating the effect of DL and RP on self-restoration and fatigue performance simultaneously from the effect of adding polymer into NA.	No	No	No	Yes
Assessing the effect of adding polymer into NA, DL, and RP on self-restoration and fatigue performance simultaneously.	No	Yes	Yes	Yes

## Data Availability

Data are contained within the article.

## Supplementary Materials

The following supporting information can be downloaded at: https://www.mdpi.com/article/10.3390/polym16192782/s1, Figure S1: DCCs related to LASH tests of all bitumen at 25% of $S_{f}$: (a) RP1; (b) RP5; (c) RP15; (d) RP30; Figure S2: DCCs related to LASH tests of all bitumen at 50% of $S_{f}$: (a) RP1; (b) RP5; (c) RP15; (d) RP30; Figure S3: DCCs related to LASH tests of all bitumen at 75% of $S_{f}$: (a) RP1; (b) RP5; (c) RP15; (d) RP30; Figure S4: DCCs related to LASH tests of all bitumen at 125% of $S_{f}$: (a) RP1; (b) RP5; (c) RP15; (d) RP30; Figure S5: DCCs of bitumen linked with cLAS test results; Figure S6: DCCs of NA related to LASH (25%, 50%, 75%, and 125% of Sf) at: (a) RP1; (b) RP5; (c) RP15; (d) RP 30 (including the DCC of cLAS as reference); Figure S7: DCCs of STPB0.5 related to LASH (25%, 50%, 75%, and 125% of Sf) at: (a) RP1; (b) RP5; (c) RP15; (d) RP 30 (including the DCC of cLAS as reference); Figure S8: DCCs of STPB1.0 related to LASH (25%, 50%, 75%, and 125% of Sf) at: (a) RP1; (b) RP5; (c) RP15; (d) RP 30 (including the DCC of cLAS as reference); Figure S9: DCCs of STPB1.5 related to LASH (25%, 50%, 75%, and 125% of Sf) at: (a) RP1; (b) RP5; (c) RP15; (d) RP 30 (including the DCC of cLAS as reference); Figure S10: DCCs of IPAB0.5 related to LASH (25%, 50%, 75%, and 125% of Sf) at: (a) RP1; (b) RP5; (c) RP15; (d) RP 30 (including the DCC of cLAS as reference); Figure S11: DCCs of IPAB1.0 related to LASH (25%, 50%, 75%, and 125% of Sf) at: (a) RP1; (b) RP5; (c) RP15; (d) RP 30 (including the DCC of cLAS as reference); Figure S12: DCCs of IPAB1.0 related to LASH (25%, 50%, 75%, and 125% of Sf) at: (a) RP1; (b) RP5; (c) RP15; (d) RP 30 (including the DCC of cLAS as reference); Figure S13: DCCs of IPAB1.0 related to LASH (25%, 50%, 75%, and 125% of Sf) at: (a) RP1; (b) RP5; (c) RP15; (d) RP 30 (including the DCC of cLAS as reference).

## Abbreviations

**Abbreviations**	**Meanings**
LAS	Linear amplitude sweep test
cLAS	Continuous linear amplitude sweep test
RP	Rest period
RP1	Rest period of 1 min
RP5	Rest period of 5 min
RP15	Rest period of 15 min
RP30	Rest period of 30 min
SPB	Self-healing polymer-modified bitumen
DL	Damage level
LASH	Linear amplitude sweep test with rest period
VECD	Viscoelastic continuum damage model
S-VECD	Simplified viscoelastic continuum damage model.
NA	Neat asphalt
STPU	Self-healing thermoplastic polyurethane
STPB	Self-healing thermoplastic polyurethane-modified bitumen
IPA1w	Self-healing poly (dimethyl siloxane) crosslinked with urea bond
IPAB	Self-healing poly (dimethyl siloxane) crosslinked with urea bond-modified bitumen
SBS	Styrene–butadiene–styrene
SBSB	Styrene–butadiene–styrene-modified bitumen
AASHTO	American Association of State Highway and Transportation official
DCC	Damage characteristic curve
PSE	Pseudo-strain energy
STPB0.5	0.5 wt% of self-healing thermoplastic polyurethane mixed with neat asphalt
STPB1.0	1.0 wt% of self-healing thermoplastic polyurethane mixed with neat asphalt
STPB1.5	1.5 wt% of self-healing thermoplastic polyurethane mixed with neat asphalt
IPAB0.5	0.5 wt% of self-healing poly (dimethyl siloxane) crosslinked with urea bond mixed with neat asphalt
IPAB1.0	1.0 wt% of self-healing poly (dimethyl siloxane) crosslinked with urea bond mixed with neat asphalt
IPAB1.5	1.5 wt% of self-healing poly (dimethyl siloxane) crosslinked with urea bond mixed with neat asphalt
RTFO	Rolling thin film oven test
PAV	Pressurized aging vessel test
PG	Performance grade
TPC	Total potential cohesion
SPC	Stored potential cohesion
RPC	Released potential cohesion
PAV.NA	Long-term aged neat asphalt
PAV.STPB0.5	Long-term aged 0.5 wt% of self-healing thermoplastic polyurethane mixed with neat asphalt
PAV.STPB1.0	Long-term aged 1.0 wt% of self-healing thermoplastic polyurethane mixed with neat asphalt
PAV.STPB1.5	Long-term aged 1.5 wt% of self-healing thermoplastic polyurethane mixed with neat asphalt
PAV.IPAB0.5	Long-term aged 0.5 wt% of self-healing poly (dimethyl siloxane) crosslinked with urea bond mixed with neat asphalt
PAV.IPAB1.0	Long-term aged 1.0 wt% of self-healing poly (dimethyl siloxane) crosslinked with urea bond mixed with neat asphalt
PAV.IPAB1.5	Long-term aged 1.5 wt% of self-healing poly (dimethyl siloxane) crosslinked with urea bond mixed with neat asphalt
PAV.SBSB	Long-term aged styrene–butadiene–styrene-modified bitumen
USA	United States of America
