# Supplementary material for: Proposal for a New Method for Evaluating Polymer-Modified Bitumen Fatigue and Self-Restoration Performances Considering the Whole Damage Characteristic Curve
Source: Polymers (Basel). 2024 Sep 30;16(19):2782. doi: 10.3390/polym16192782 (PMC11479077; doi:10.3390/polym16192782)
Supplement: Supplementary file 1 [file polymers-16-02782-s001.zip › Supplementary materials.pdf]

Supplementary materials.

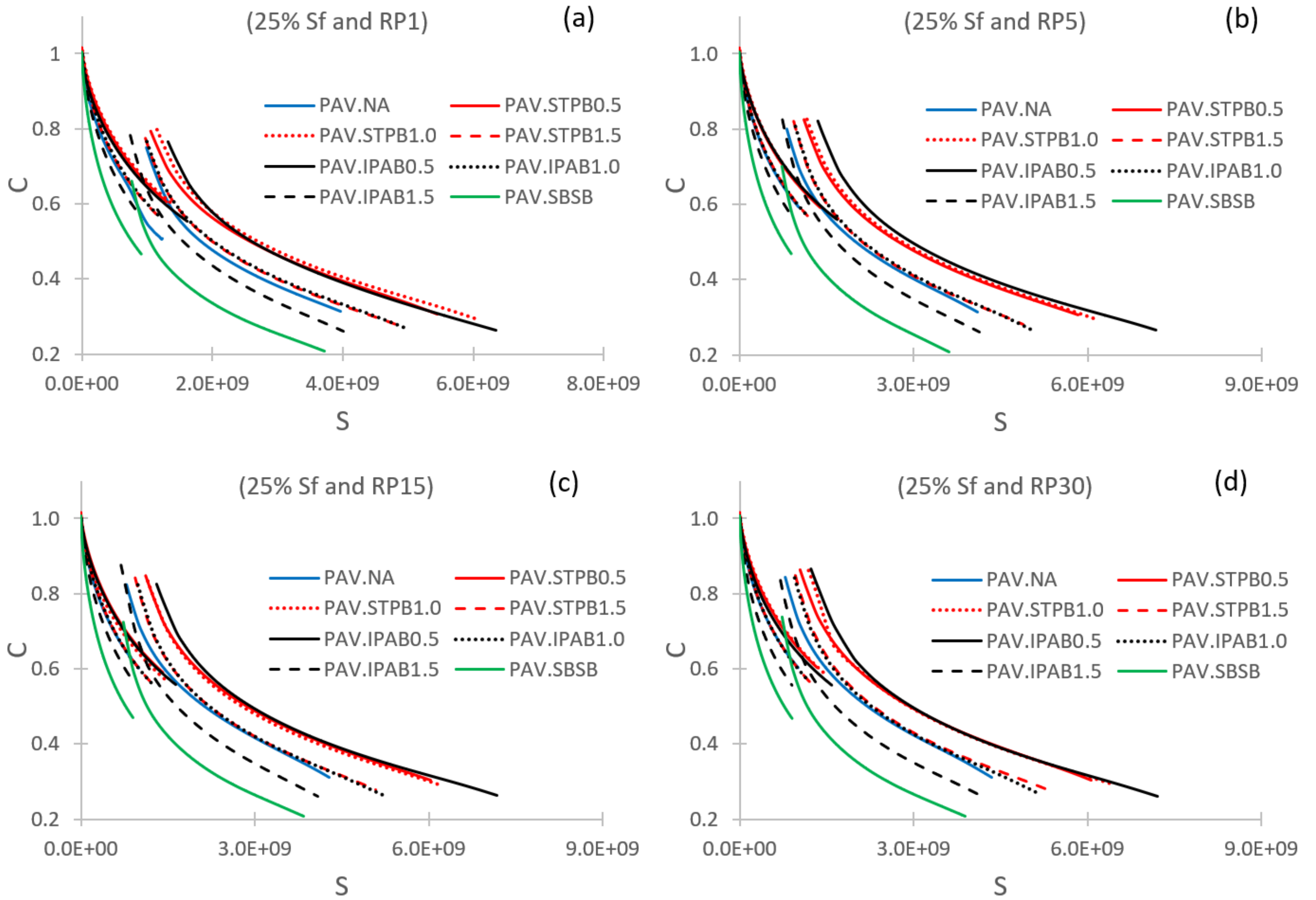

Figure S 1. DCCs related to LASH tests of all bitumens at 25% of S_f: (a) RP1; (b) RP5; (c) RP15; (d) RP30.

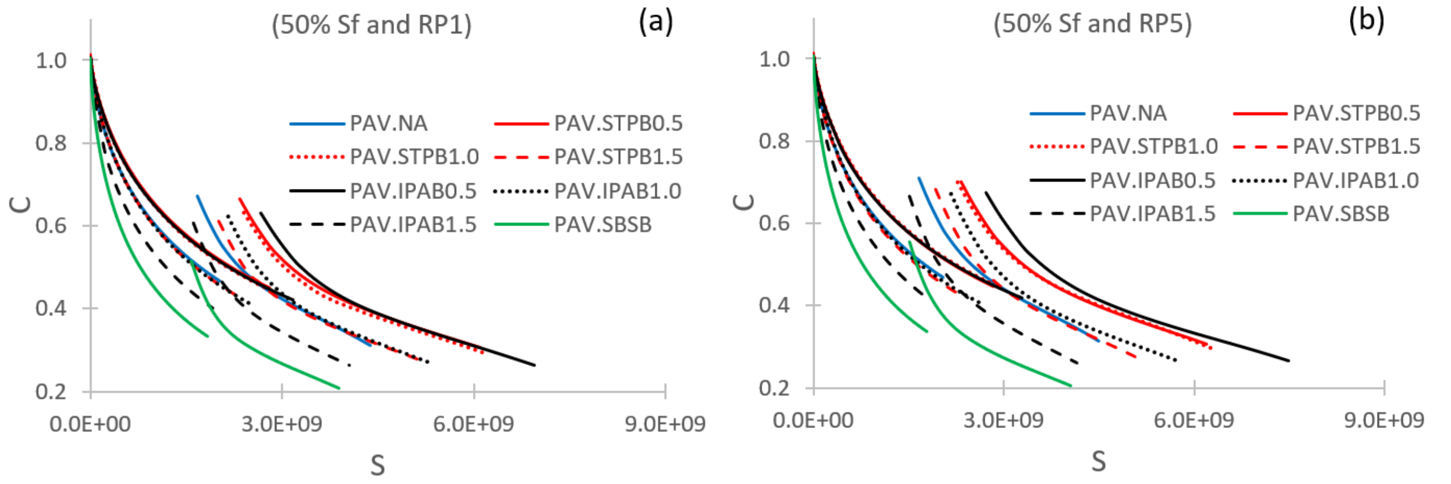

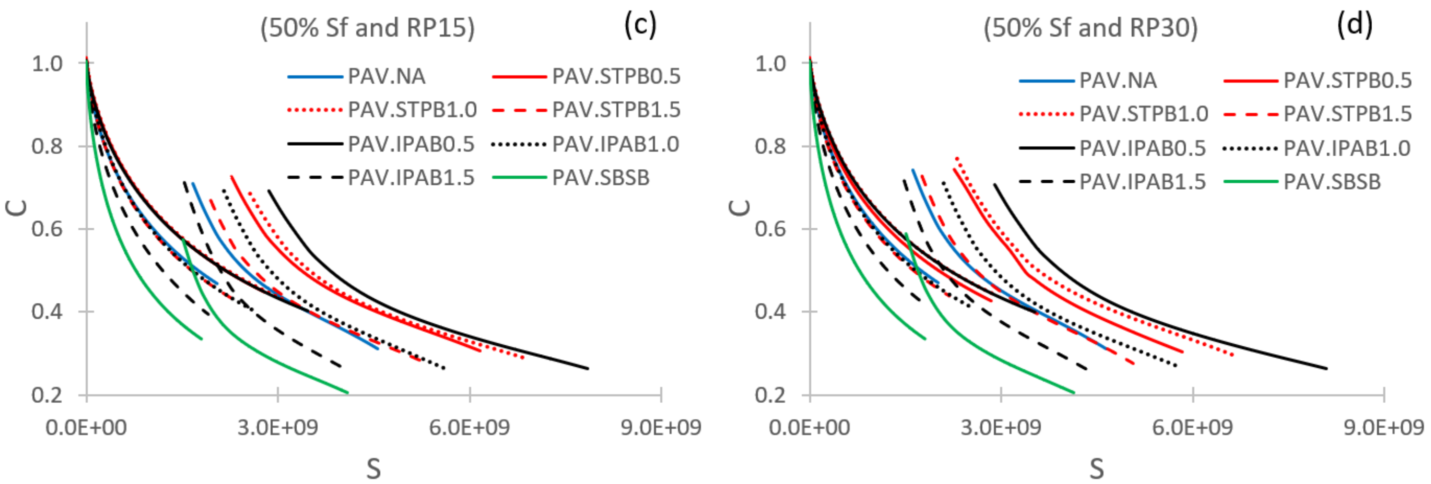

Figure S 2. DCCs related to LASH tests of all bitumens at 50% of S_f: (a) RP1; (b) RP5; (c) RP15; (d) RP30.

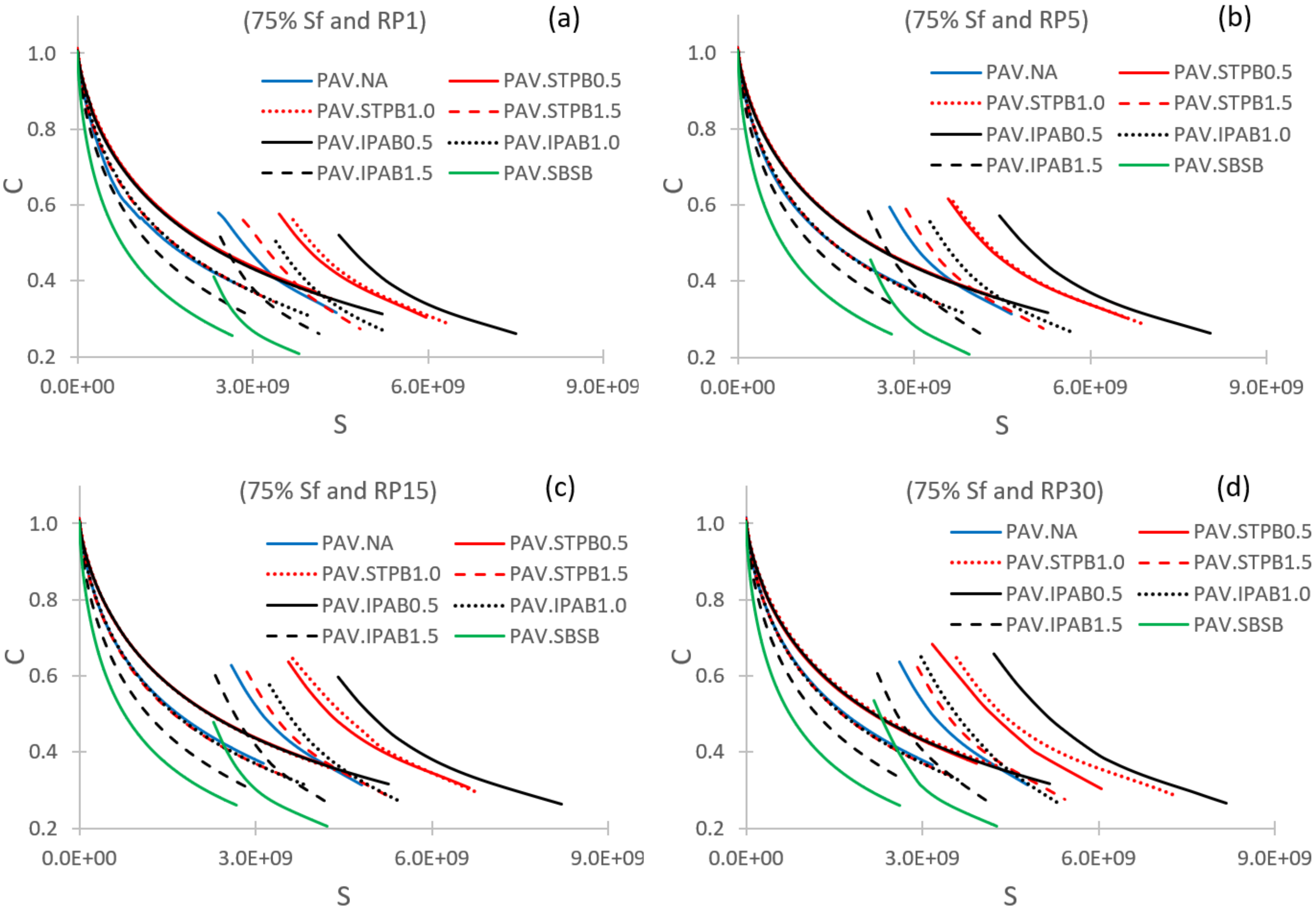

Figure S 3. DCCs related to LASH tests of all bitumens at 75% of S_f: (a) RP1; (b) RP5; (c) RP15; (d) RP30.

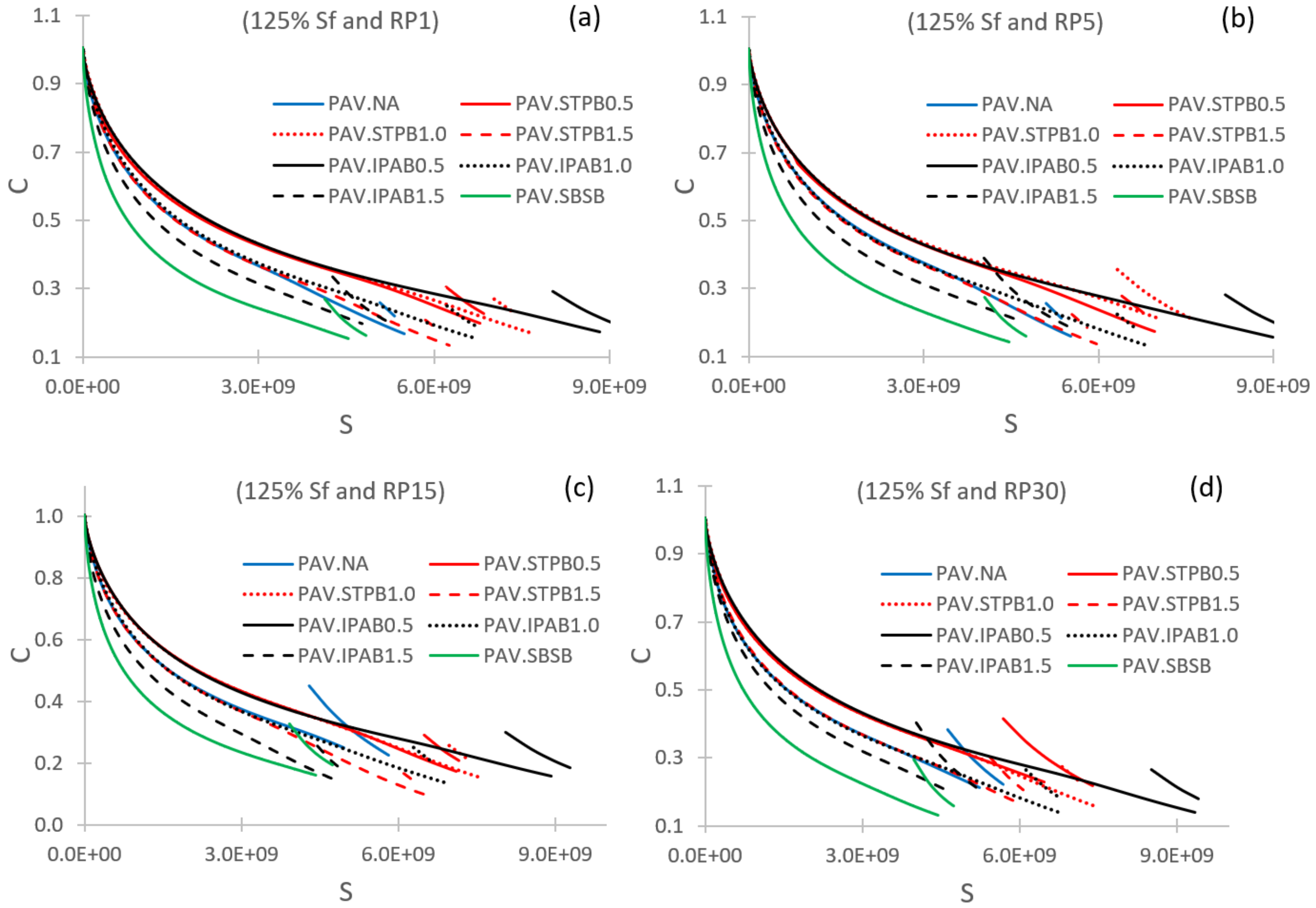

Figure S 4. DCCs related to LASH tests of all bitumens at 125% of S_f: (a) RP1; (b) RP5; (c) RP15; (d) RP30.

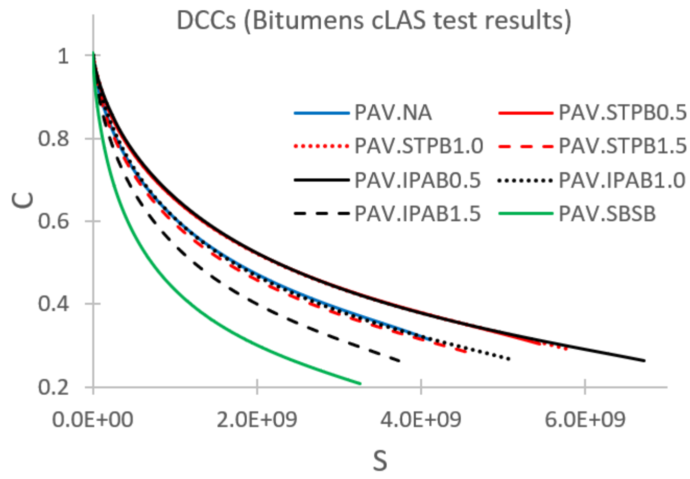

Figure S 5 DCCs of bitumens linked with cLAS test results.

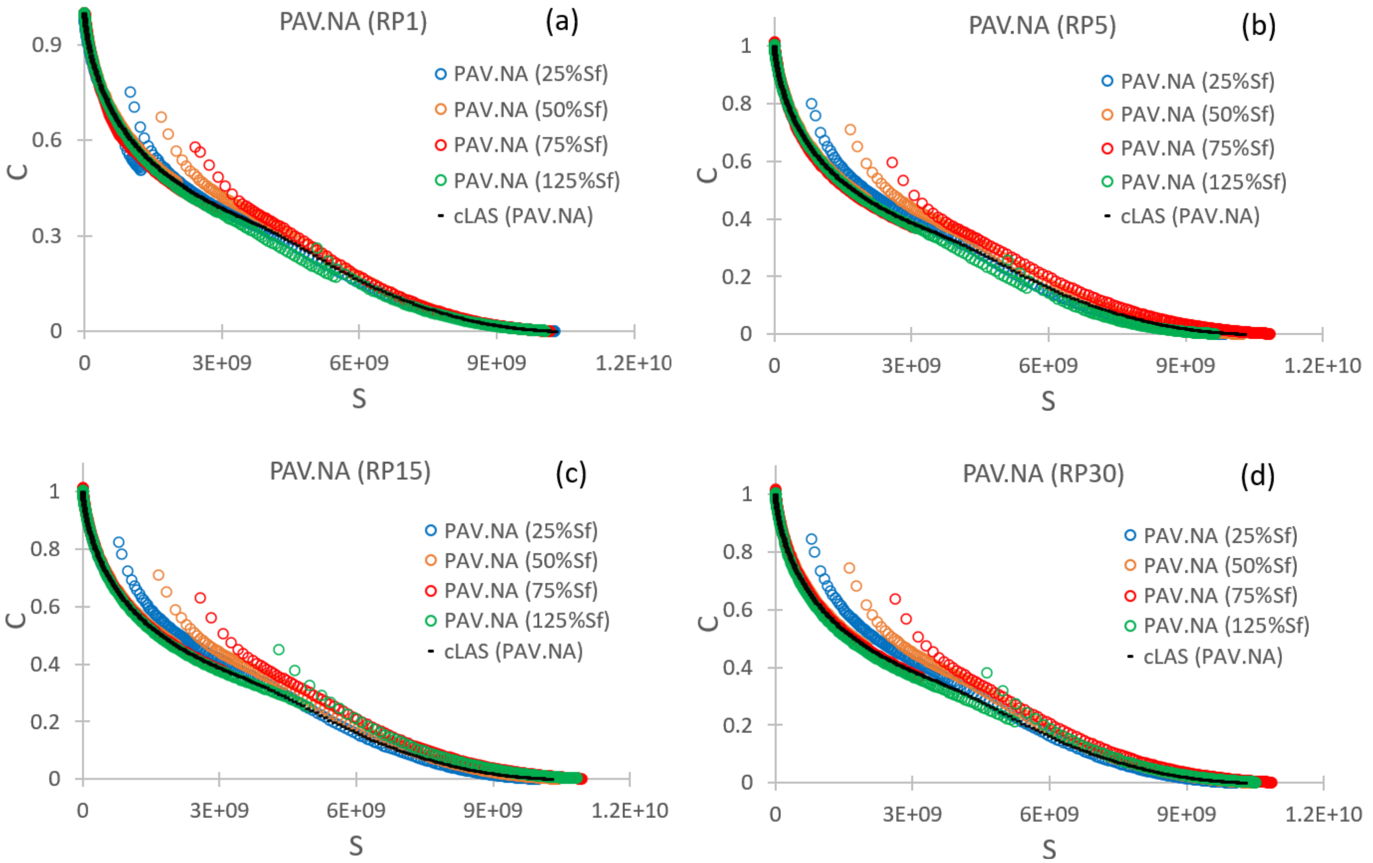

Figure S 6 DCCs of NA related to LASH (25%, 50%, 75%, and 125% of Sf) at: (a) RP1; (b) RP5; (c) RP15; (d) RP 30 (including the DCC of cLAS as reference).

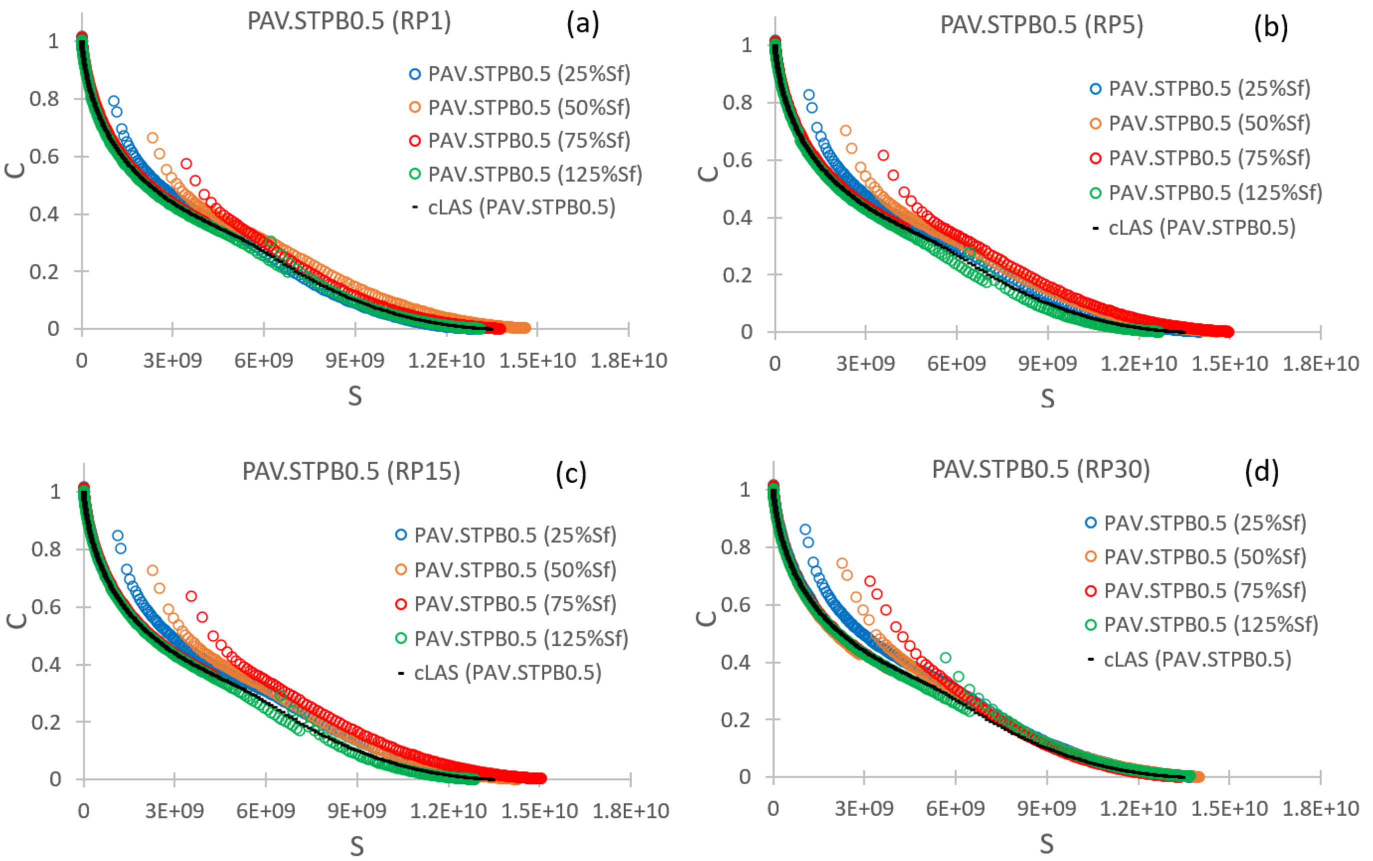

Figure S 7 DCCs of STPB0.5 related to LASH (25%, 50%, 75%, and 125% of Sf) at: (a) RP1; (b) RP5; (c) RP15; (d) RP 30 (including the DCC of cLAS as reference).

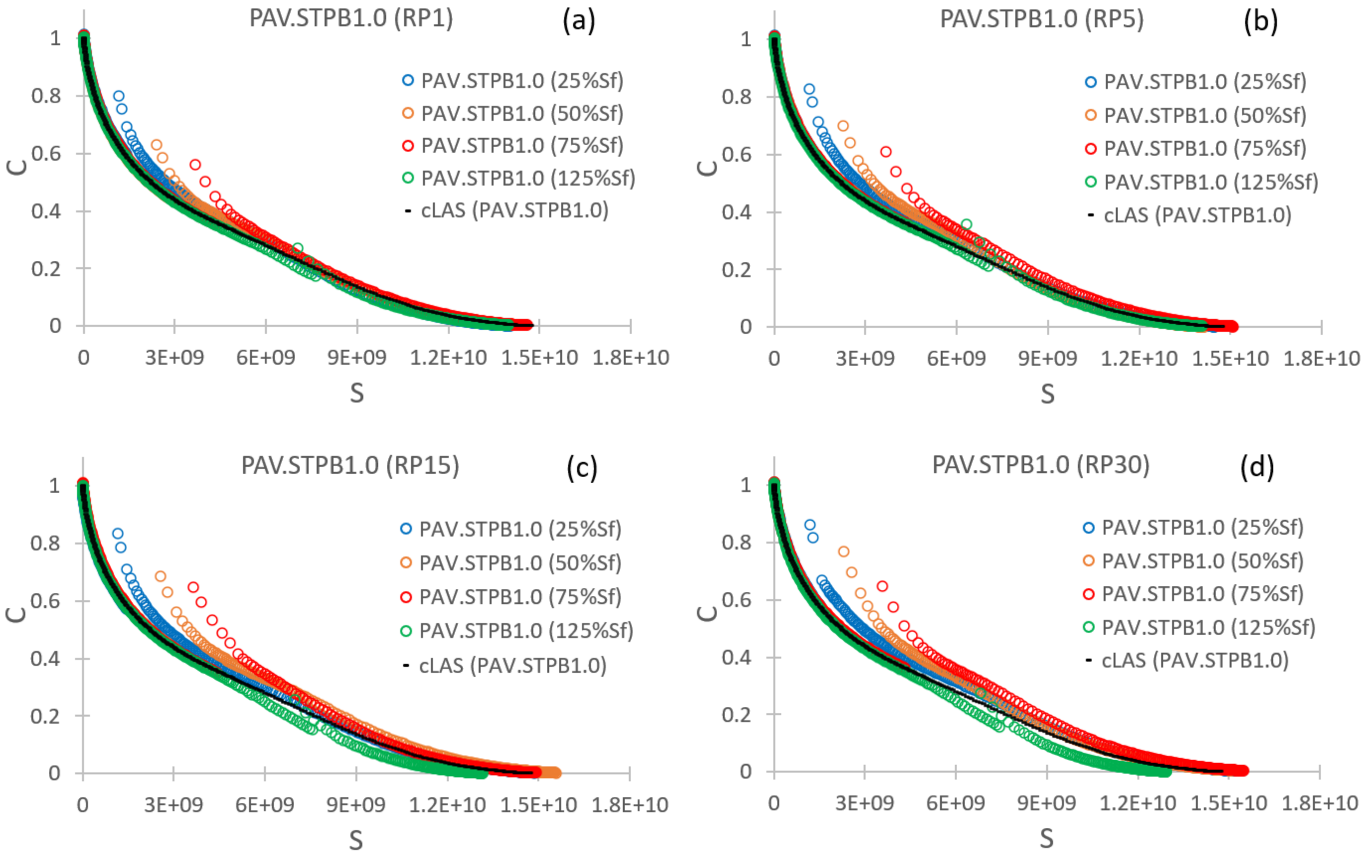

Figure S 8 DCCs of STPB1.0 related to LASH (25%, 50%, 75%, and 125% of Sf) at: (a) RP1; (b) RP5; (c) RP15; (d) RP 30 (including the DCC of cLAS as reference).

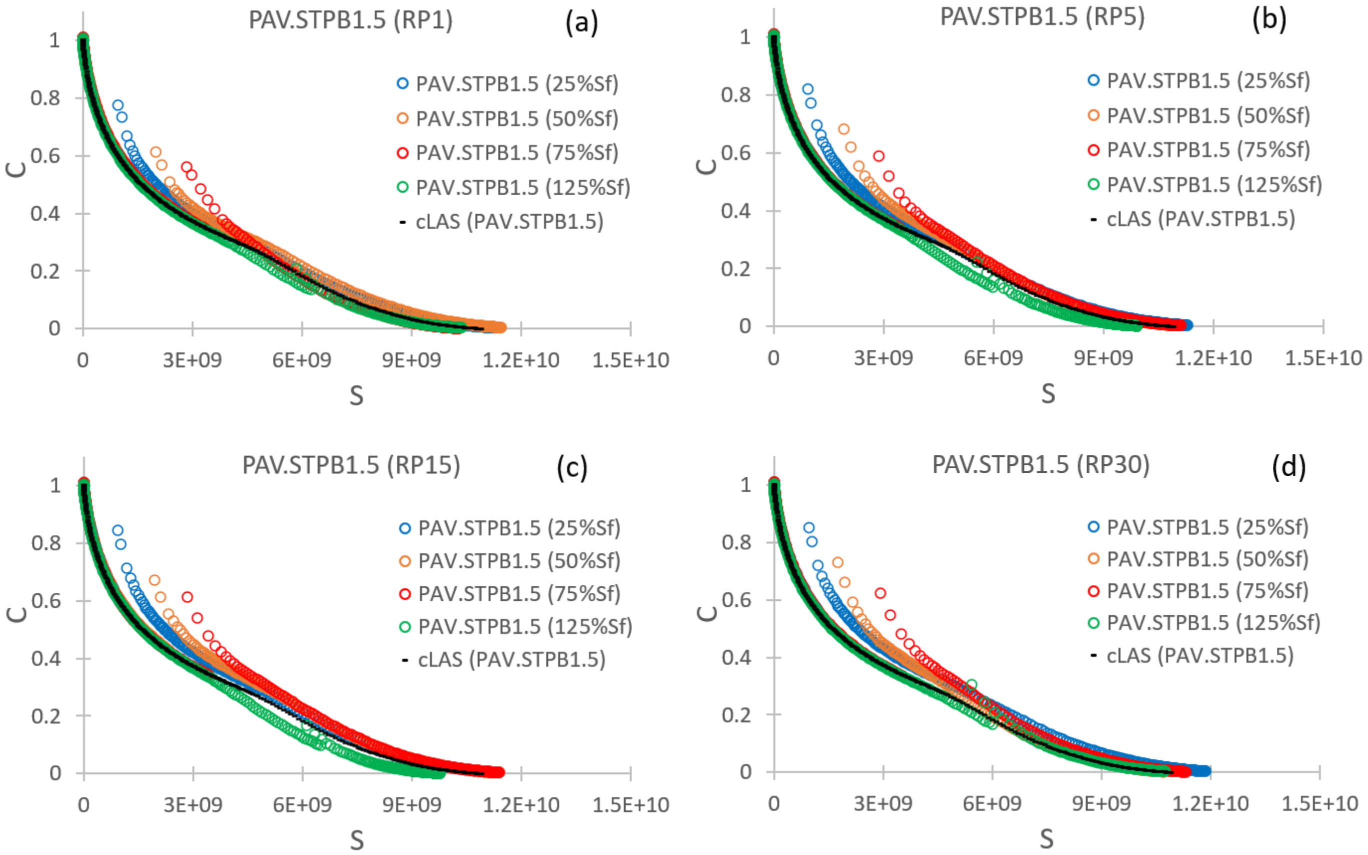

Figure S 9 DCCs of STPB1.5 related to LASH (25%, 50%, 75%, and 125% of Sf) at: (a) RP1; (b) RP5; (c) RP15; (d) RP 30 (including the DCC of cLAS as reference).

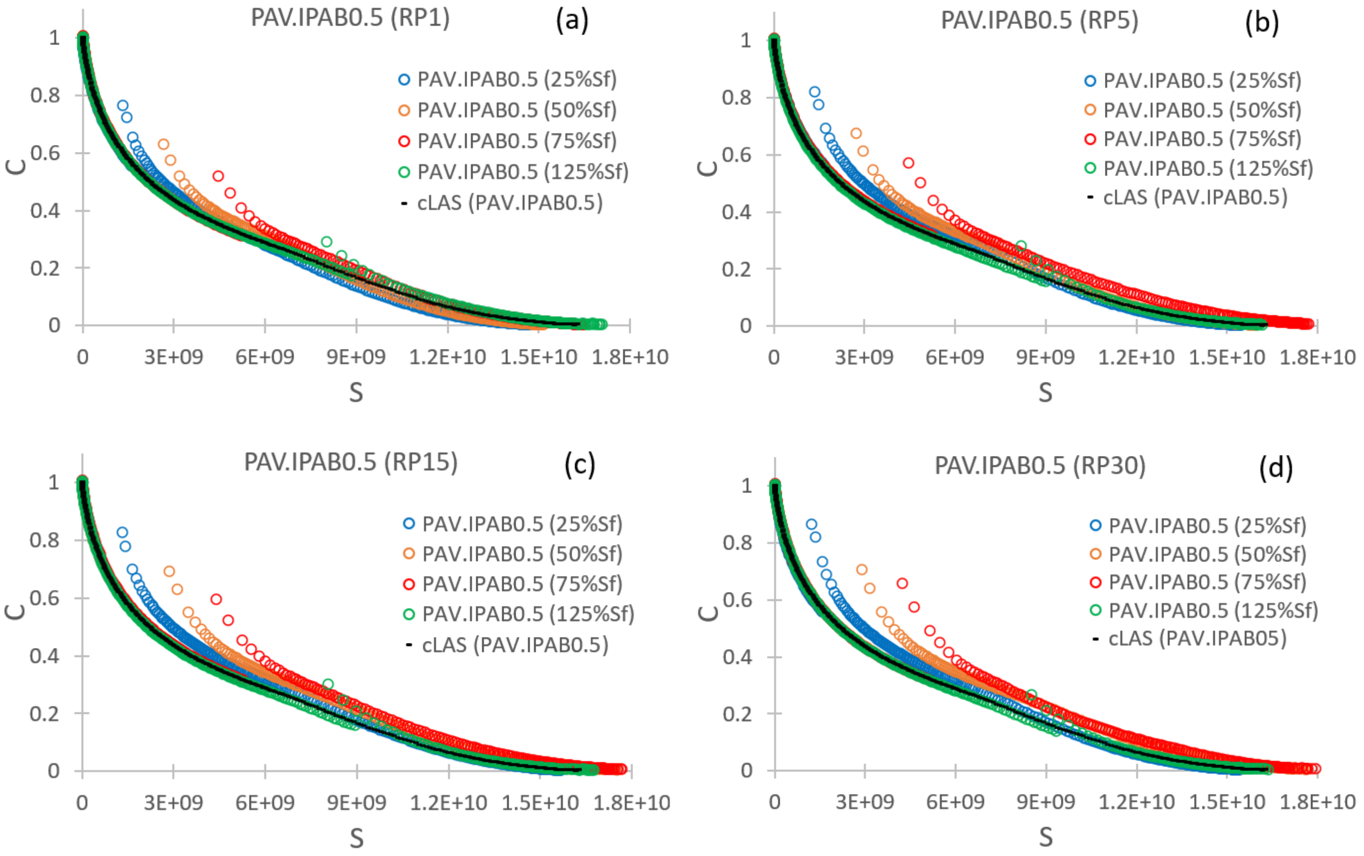

Figure S 10 DCCs of IPAB0.5 related to LASH (25%, 50%, 75%, and 125% of Sf) at: (a) RP1; (b) RP5; (c) RP15; (d) RP 30 (including the DCC of cLAS as reference).

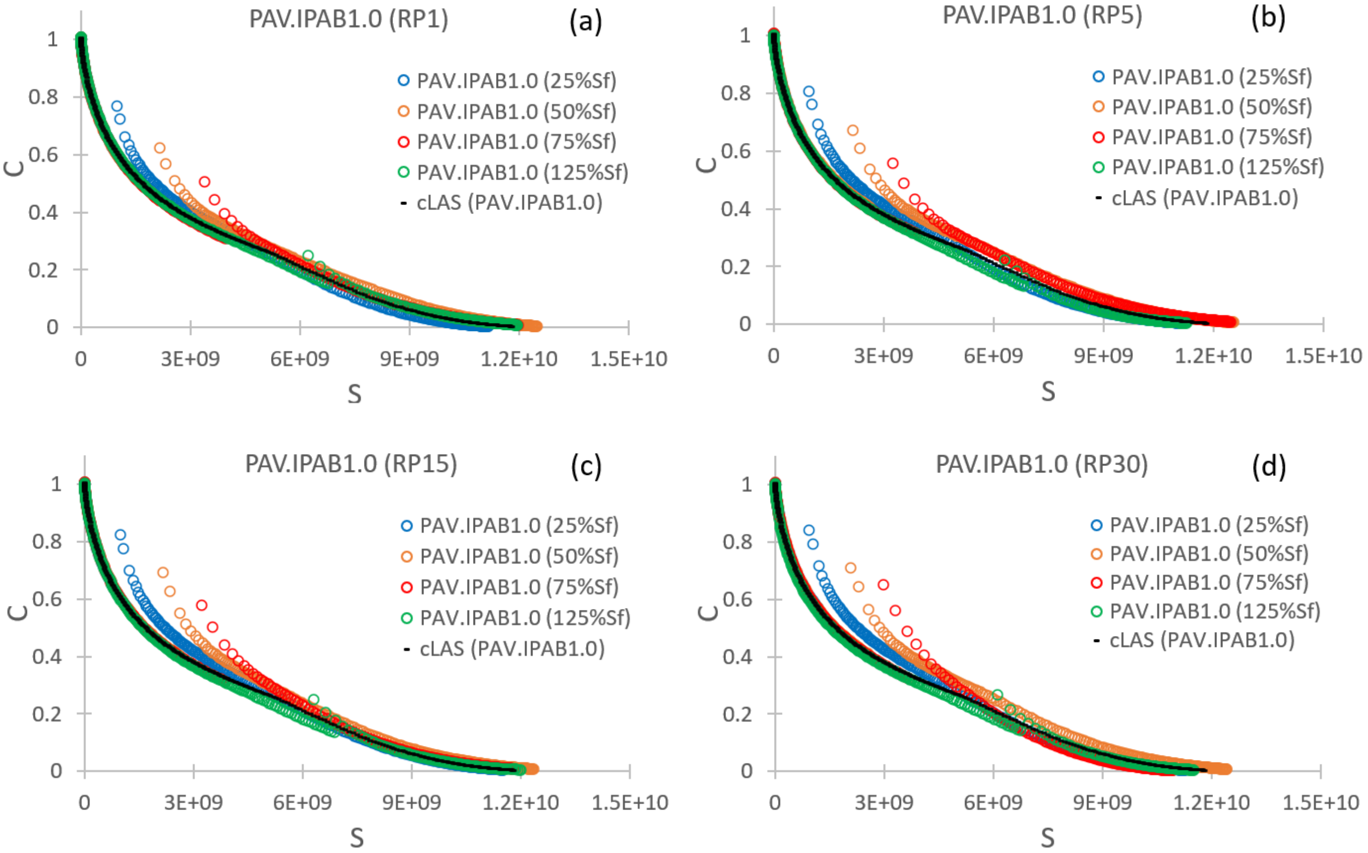

Figure S 11 DCCs of IPAB1.0 related to LASH (25%, 50%, 75%, and 125% of Sf) at: (a) RP1; (b) RP5; (c) RP15; (d) RP 30 (including the DCC of cLAS as reference).

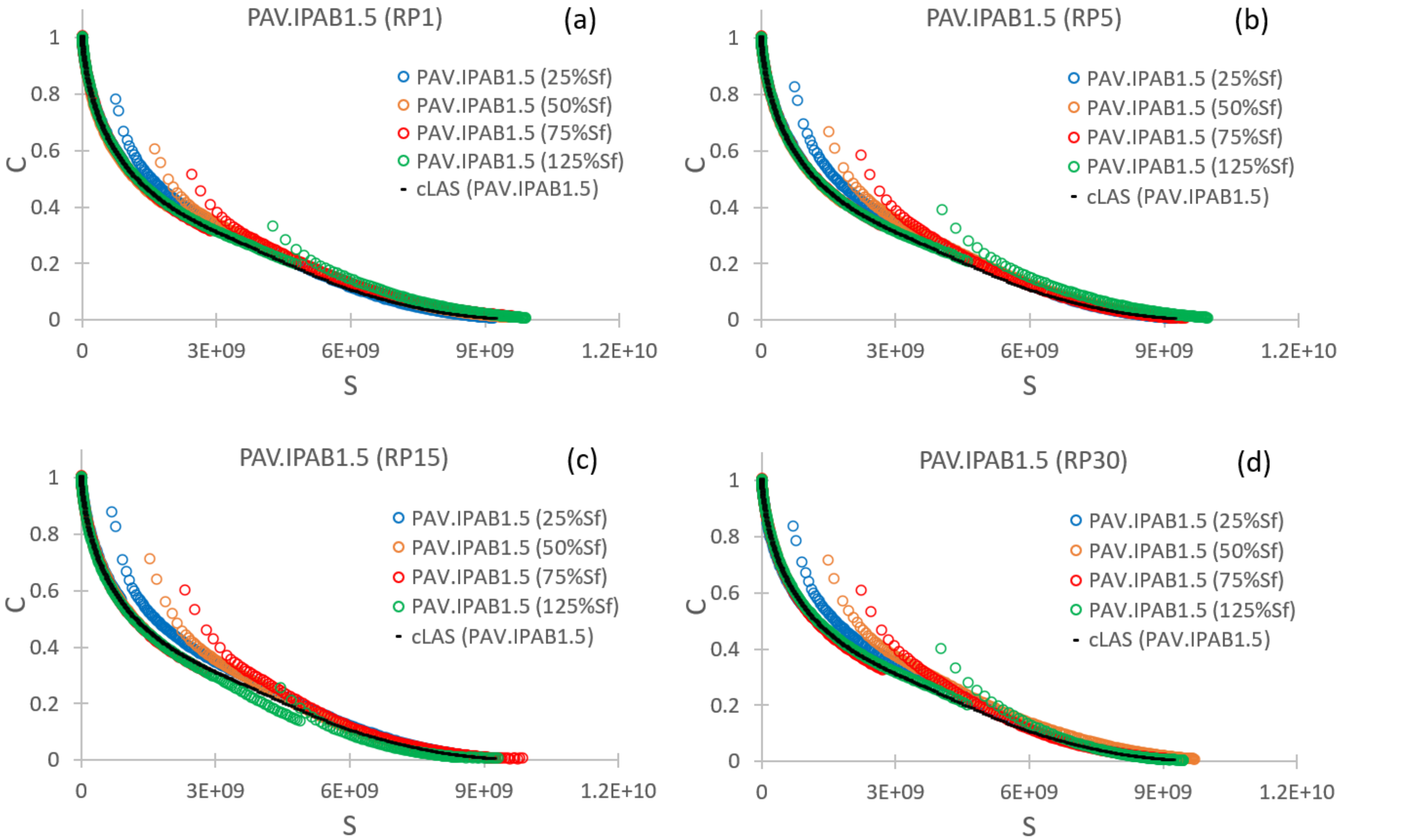

Figure S 12 DCCs of IPAB1.0 related to LASH (25%, 50%, 75%, and 125% of Sf) at: (a) RP1; (b) RP5; (c) RP15; (d) RP 30 (including the DCC of cLAS as reference).

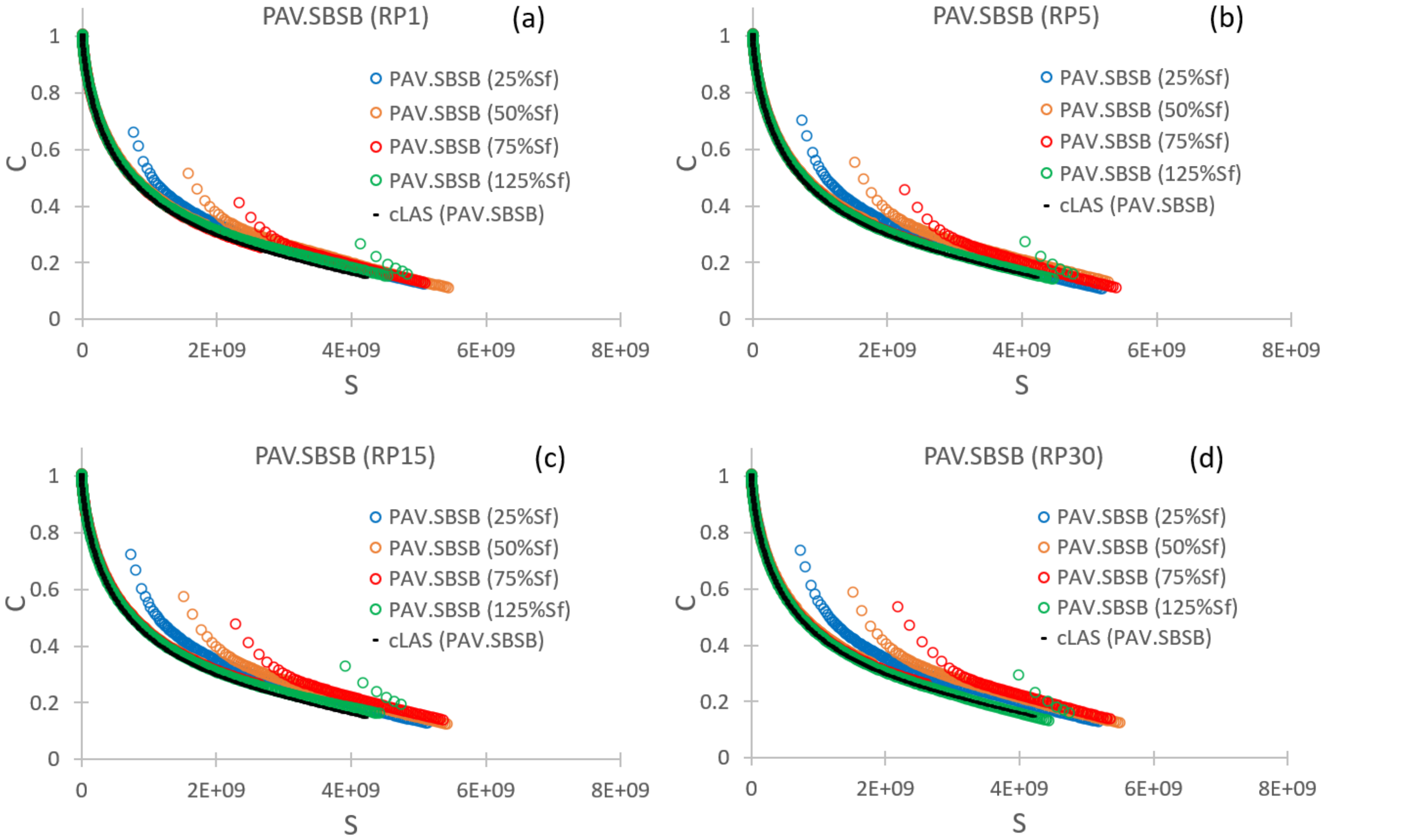

Figure S 13 DCCs of IPAB1.0 related to LASH (25%, 50%, 75%, and 125% of Sf) at: (a) RP1; (b) RP5; (c) RP15; (d) RP 30 (including the DCC of cLAS as reference).
